# Small molecule- and cell contact-inducible systems for controlling expression and differentiation in mouse embryonic stem cells

**DOI:** 10.1242/dev.204505

**Published:** 2025-06-10

**Authors:** Sarah S. Soliman, Devan H. Shah, Hana El-Samad, Zara Y. Weinberg

**Affiliations:** ^1^ Department of Biochemistry and Biophysics, University of California, San Francisco, CA 94158, USA; ^2^ UC Berkeley-UCSF Graduate Program in Bioengineering, University of California, Berkeley, CA 94720-1762, USA; ^3^ Cell Design Institute, University of California, San Francisco, CA 94158, USA; ^4^ Chan-Zuckerberg Biohub, San Francisco, CA 94158, USA; ^5^ Altos Labs, Redwood City, CA 94063, USA

**Keywords:** Synthetic development, Gene expression, Differentiation, Stem cells, Cell engineering, Inducible systems

## Abstract

Synthetic developmental biology uses engineering approaches to understand multicellularity with goals ranging from recapitulating development to building synthetic organisms. Current approaches include engineering multicellular patterning, controlling differentiation and implementing cooperative cellular behaviors in model systems. Synthetic biology enables these pursuits by providing tools to control cell behavior. Mouse embryonic stem cells (mESCs) offer a well-studied and genetically tractable pluripotent model for pursuing synthetic development questions. However, there is minimal characterization of existing synthetic biology tools in mESCs. Here, we characterize three small molecule- and two cell contact-inducible systems for gene expression in and differentiation of mESCs. We show that small molecule- and cell contact-inducible systems work reliably and efficiently for controlling expression of arbitrary genetic payloads. We identify how these systems function differently across model differentiations. Furthermore, we show that these systems can drive direct differentiation of mESCs into neurons. Each of these systems can be used on their own or in combination, raising many possibilities for studying developmental principles with high precision.

## Teaser

**Highlighted Article:** Identification and characterization of GAL4 upstream activation sequence-based inducible systems for use with either small molecule or juxtacrine induction to expand the cell engineering toolkit in mESCs.

## INTRODUCTION

Synthetic developmental biology seeks to decipher the principles of developmental processes using a build-to-understand approach (Elowitz and Lim, 2010). This entails building synthetic systems that recapitulate multicellular developmental paradigms such as differentiation, patterning and cooperative cellular behaviors (Davies, 2017; Ebrahimkhani and Ebisuya, 2019; Martínez-Ara et al., 2022; Santorelli et al., 2019; Toda et al., 2019; Todhunter et al., 2015). The field of synthetic developmental biology is still nascent, but holds immense promise, spanning from controlling tissue development in a dish to engineering fully synthetic tissues and building synthetic organisms (Johnson et al., 2017; McNamara et al., 2023; Schlissel and Li, 2020; Trentesaux et al., 2023; Velazquez et al., 2018).

Synthetic biology tools are essential for synthetic development and have been used in practically every landmark study in this field (Ebrahimkhani and Ebisuya, 2019; Johnson et al., 2017; McNamara et al., 2023; Santorelli et al., 2019; Schlissel and Li, 2020; Toda et al., 2019; Velazquez et al., 2018). Synthetic biology has produced a repertoire of sophisticated tools for controlling cellular behavior that enables customized responses to arbitrary stimuli, including synthetic receptors that enable orthogonal signaling channels, and light- or drug-inducible systems that enable precise spatial and temporal control of cell function (Johnson and Toettcher, 2018; Kowalik and Chen, 2017; Li et al., 2022; Malaguti et al., 2024; Meyer et al., 2023; Morsut et al., 2016; Toettcher et al., 2011; Zhu et al., 2022). Cell engineering has been advanced through packaging these tools into robust modular toolkits, such as the Mammalian Toolkit (MTK) (Fonseca et al., 2019). Adapting and evaluating these tools in developmental model systems will facilitate the goals of synthetic development.

When evaluating new tools in a synthetic developmental context, reprogramming is an accessible and well-studied testbed for applying synthetic biology tools (Wang et al., 2020). Overexpression of transcription factors has been heavily used in the field of stem cell differentiation (Briggs et al., 2017; Ng et al., 2021; Oh and Jang, 2019; Peterson, 1993). For example, multiple studies have demonstrated that the proneural transcription factor neurogenin 2 (Ngn2) is necessary and sufficient to specify glutamatergic neuronal identity (Flitsch et al., 2020; Thoma et al., 2012). Efforts to control transcription factor overexpression in stem cells have primarily used the artificial transcriptional regulator TetR which is derived from microbial transcription factor and induced by the small molecule antibiotic doxycycline (Gossen and Bujard, 1992; Gossen et al., 1995). In recent examples, the synthetic receptor synNotch has been used in mESCs (Malaguti et al., 2022) and in human embryonic stem cells (hESCs) (Lee et al., 2023) to drive neuronal differentiation.

Expanding the available tools for cell engineering enables more complex control that would allow us to better model developmental systems. To address this, we sought to expand the synthetic developmental toolbox by engineering synthetic biology tools into embryonic stem cells. While recent approaches in synthetic biology have elegantly recapitulated development in simple cellular systems, additional approaches are needed to fully realize the potential of synthetic developmental biology. Robust modular tools for engineering cellular behavior have had important impacts in yeast and mammalian cell engineering, and we sought to bring those to stem cells. Here, we describe an approach for rapid engineering of multiple inducible systems into mouse embryonic stem cells (mESCs). We show that these systems work reliably and efficiently in controlling expression of arbitrary genetic payloads. We identify how these systems function differently across model differentiations. Furthermore, we show that some of these systems are capable of driving direct differentiation of mESCs into neuron-like cells, opening up questions about the quantitative requirements of these differentiation programs. The components and approaches we describe can readily be used on their own or in combination to engineer mESCs in synthetic development studies and further expand the toolkit for studying developmental principles with high precision.

## RESULTS

### Small molecule synthetic transcription factor systems induce protein expression in mESCs

We first sought to build and test small molecule-inducible synthetic transcription factor systems in mESCs. Inspired by the clinically relevant synthetic zinc-finger transcription regulators (synZiFTRs) system (Li et al., 2022), we adapted three different drug-inducible control switches for use in mESCs. The first of these drugs is 4-hydroxytamoxifen (4OHT), which selectively modulates the nuclear availability of molecules fused to a sensitized variant of the human estrogen receptor Ert2 (Feil et al., 1996; Indra et al., 1999). This 4OHT/Ert2 system has been widely used previously in mESCs and developmental biology in general, especially coupled with the Cre recombinase (Feil et al., 1996; Indra et al., 1999; Schwenk et al., 1998; Vallier et al., 2001; Zhang et al., 1996). The second drug is abscisic acid (ABA), which mediates conditional binding of complementary protein fragments (ABI and Pyl) from the ABA stress response pathway to reconstitute an active transcription factor (Liang et al., 2011). The third drug is grazoprevir (GZV), a protease-inhibitor that, when added, stabilizes transcription factors incorporating the hepatitis C NS3 self-cleaving protease domain (Jacobs et al., 2018; Tague et al., 2018).

**Fig. 1.**
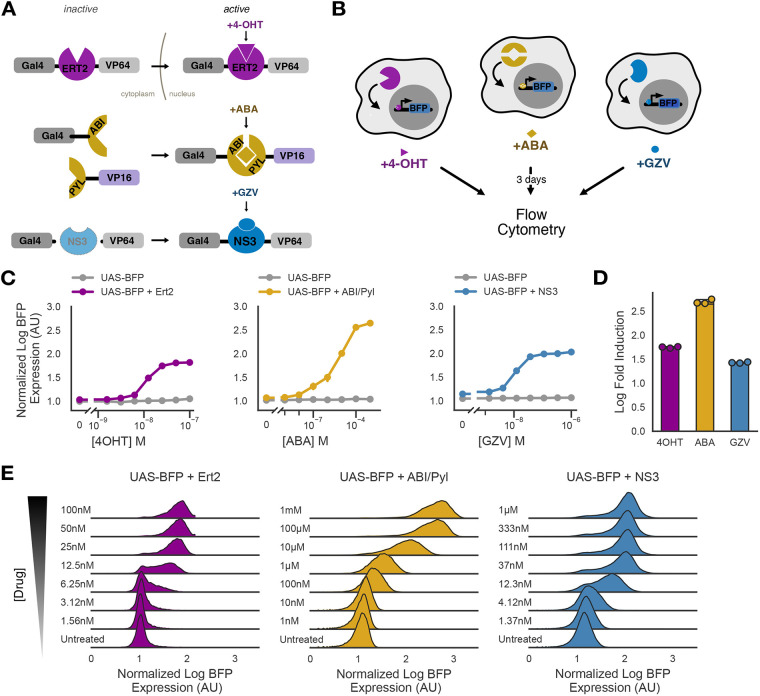
**Small molecule synthetic transcription factor systems induce protein expression in mESCs.** (A) Schematic of the mechanism of action of small molecule synthetic transcription factor systems. (B) Schematic of the small molecule synthetic transcription factor systems assay. (C) Dose-response of curves of median log BFP expression (normalized to untreated UAS-BFP cells) for each small molecule synthetic transcription factor system. (D) Median log fold induction levels for each system at maximum dose (100 nM 4OHT, 1 mM ABA or 1 µM GZV). (E) Histograms describing population density estimates of each small molecule-inducible system across doses.

To ensure orthogonality of our transcriptional system from the murine genome, we employed the GAL4 upstream activating sequence (UAS) from yeast as our inducible promoter (Kakidani and Ptashne, 1988; Webster et al., 1988). We selected a version of the GAL4 UAS that is coupled to the minimal promoter ybTATA (Hansen et al., 2014), which performs well in mammalian systems with low background activation, a high signal to noise ratio and tunability (Ede et al., 2016; Weinberg et al., 2021 preprint; Zhu et al., 2022). The GAL4 UAS-ybTATA system has not been used in mESCs and therefore it would represent an expansion to the orthogonal transcriptional regulatory elements toolkit. Although there are a multitude of orthogonal transcriptional elements [including the well described synZiFTR (Li et al., 2022) system], we selected the GAL4 UAS because of its broad compatibility with existing synthetic biology tools, including the MTK (Fonseca et al., 2019). We constructed our synthetic transcription factor system using the GAL4 DNA-binding and the transactivation domains of either VP16 (fused to ABI/Pyl) or VP64 (4X tandem VP16, fused to Ert2 and NS3) coupled to a constitutively expressed mCherry as a marker of construct expression (Fig. 1A, Fig. S1A) (Beerli et al., 1998; Sadowski et al., 1988).

To test the function of each of these systems, we used an output genetic cassette driving inducible blue fluorescent protein (mTagBFP2, BFP) downstream of a 5X tandem GAL4 UAS sequence and a ybTATA minimal promoter alongside a constitutively expressed mCitrine (Weinberg et al., 2021 preprint; Zhu et al., 2022) (Fig. S1B). We used lentiviral transduction to introduce this cassette into mESCs and then sorted a mixed population of cells based on high mCitrine expression (Fig. S2A). We will refer to these cells at UAS-BFP mESCs. We then stably integrated our synthetic transcription factor systems into the UAS-BFP mESC line by lentiviral transduction and sorted cells based on similar mCherry expression (Fig. S2B). UAS-BFP cells have very little leak, with BFP fluorescence only modestly higher than in wild-type mESCs (Fig. S2C). In the absence of inducer, UAS-BFP cells and each transcription factor-expressing subline showed minimal BFP expression or leakage at baseline (Fig. S2D).

We cultured UAS-BFP mESCs expressing each system with different concentrations of their specific inducer for 3 days, and assessed drug-dependent expression of TagBFP via flow cytometry (Fig. 1B). The three inducible systems exhibited titratable control of TagBFP output, minimal leakage relative to TagBFP-only cells and strong dynamic ranges (Fig. 1C). Each of these systems exhibited strong levels of induction (Fig. 1D). These systems exhibited different dose-dependent population distribution dynamics within mESCs. Across a titratable dose, both Ert2 and NS3 shifted individual cells between discrete ON and OFF states, while ABI/Pyl showed a continuous response in individual cells (Fig. 1E). Similar to the synZiFTRs (Li et al., 2022), these systems returned to basal levels upon removal of the inducer at maximum concentration. Specifically, Ert2, ABI/Pyl and NS3 showed varying OFF-rate kinetics, returning to basal levels at days 5, 11 and 13, respectively (Fig. S2E). Furthermore, we generated cells with a 1.5 log wider range of expression for each transcription factor system from the high-sorted cells and observed that increased TagBFP expression correlated with increased transcription factor system expression in a dose-dependent manner (Fig. S2F). These data indicate that these three small molecule synthetic transcription factor systems work reliably and efficiently to induce TagBFP expression in mESCs, suggest expression and dosing regimes for optimizing payload expression, and position these tools for use in more elaborate gene switches and in controlling more-complex genetic payloads.

For these small molecule inducers to be useful in mESCs, they should have minimal effects on pluripotency. Therefore, we characterized the effect of each small molecule on the expression of naive pluripotency markers (Nichols and Smith, 2009; Weinberger et al., 2016) in mESCs cultured in 2i. While the small molecule 4OHT has been widely used over the years in mESCs (Vallier et al., 2001; Zhang et al., 1996), ABA and GZV are less well characterized. We compared the expression of the pluripotency markers Nanog, Oct3/4 and Sox2 in wild-type mESCs with each inducer at maximum dose (100 nM 4OHT, 1 mM ABA or 1 µM GZV) incubation or with vehicle treatment (referred to as untreated) after 3 days. We saw no effect on any marker expression between untreated and treated conditions for all three drugs (Fig. S3A). These results indicate that these drugs have minimal effects on the pluripotency state of mESCs.

### Small molecule-inducible systems function across differentiation states

One of the primary reasons we selected mESCs as a target for cell engineering is their power to model a variety of differentiation paradigms. The value of the inducible systems we implemented in these cells is, in part, dependent on the ability of these systems to function during and after model differentiations. Differentiation is coupled to fundamental changes in cell state, such as protein synthesis rate (Buszczak et al., 2014) and chromatin organization (Dixon et al., 2015). In addition to changes in the intrinsic abilities of different cell types to produce protein and regulate specific genes, transgene expression can be susceptible to epigenetic silencing across cell types (Dorer and Henikoff, 1997; Kwaks and Otte, 2006). To test the ability of our engineered mESC small molecule-inducible systems to sustain transgene expression across differentiation, we differentiated our lines into multiple lineages using previously described protocols (Semrau et al., 2017; Yang et al., 2019).

**Fig. 2.**
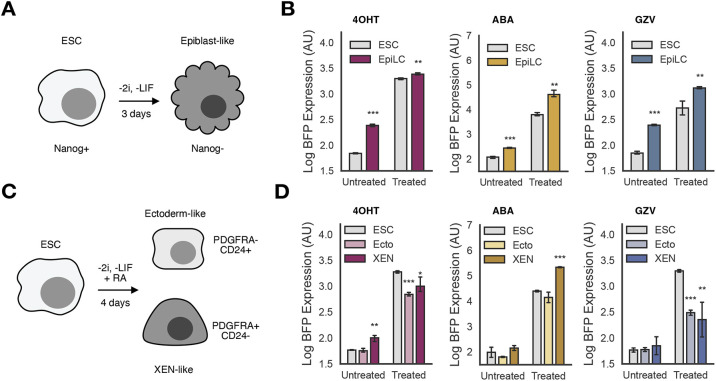
**Small molecule-inducible systems function across differentiation states.** (A) Schematic of the differentiation assay for epiblast-like cells (EpiLCs) from embryonic stem cells (ESCs). Each small molecule-inducible mESC line was cultured with or without drug at maximum dose (100 nM 4OHT, 1 mM ABA or 1 µM GZV) and in either 2i or SFES (-2i, -LIF). EpiLCs were assessed via Nanog− marker expression. (B) Comparison of BFP expression for each small molecule-inducible system in treated versus untreated for ESCs cultured in 2i and for EpiLCs. (C) Schematic of the differentiation assay for ectoderm-like (Ecto) and extraembryonic endoderm-like (XEN) cells. Each small molecule-inducible mESC line was cultured with or without drug at maximum dose (100 nM 4OHT, 1 mM ABA or 1 µM GZV) and in either 2i media or SFES (-2i and -LIF) with 0.25 μM retinoic acid. Ectoderm-like cells were identified via PDGFRA− and CD24+ marker expression; conversely, XEN-like cells were identified via PDGFRA+ and CD24− marker expression. (D) Comparison of BFP expression each small molecule-inducible system in treated versus untreated ESCs cultured in 2i, and Ecto and XEN cells. Statistical comparisons are pairwise *t*-tests to undifferentiated ESCs at each dose, ns, *P*>0.05; *P<0.05; **P<0.01; ***P<0.001.

We differentiated each of our small molecule-inducible mESC lines into epiblast-like cells (EpiLCs) by culturing mESCs in serum-free ES media (Mulas et al., 2019; Yang et al., 2019) without 2i and LIF for 3 days (Fig. 2A). We stained the differentiated cells for Nanog expression (Fig. S4A) and identified Nanog-negative cells as EpiLCs. We then compared BFP expression in cells treated with vehicle or a saturating dose of each inducer (100 nM 4OHT, 1 mM ABA or 1 µM GZV) to BFP levels in the same cell line when cultured for the same amount of time in 2i (Fig. 2B). For all three inducers, both basal (untreated) and induced (treated) BFP expression was significantly higher in EpiLCs compared to undifferentiated cells of the same line. We additionally evaluated the effect of each inducer on differentiation efficiency (Fig. S4B) by calculating the fraction of all cells identified as EpiLCs in our final sample. Ethanol, the vehicle for both 4OHT and ABA, decreased EpiLC differentiation when compared to control. DMSO had no effect on differentiation on its own. In the treated condition, 4OHT had a dramatic effect on differentiation, significantly decreasing the fraction of EpiLCs compared to the vehicle condition. ABA had no effect on differentiation greater than its vehicle effect. GZV modestly but significantly decreased EpiLC production compared to vehicle.

We repeated this experiment but with a previously described (Semrau et al., 2017) differentiation in retinoic acid (RA) that can produce ectoderm-like (Ecto) and extra-embryonic endoderm (XEN)-like cells (Fig. 2C). Cells were cultured for 4 days in SFES with 0.25 µM RA and either vehicle or the same concentration of each inducer used above. Cells were stained for CD24 (a previously reported marker for Ecto cells; Semrau et al., 2017) and PDGFRA (a previously reported marker for XEN cells; Semrau et al., 2017), and were identified as each cell type if they were positive for only one of the markers (Fig. S4C). When Ecto and XEN cells are compared to mESCs cultured in 2i with or without inducer for the same amount of time, a complex effect of cell identity was observed on inducer function (Fig. 2D). Expression levels of the transcription factors changed modestly but significantly in differentiated cells, as assessed by mCherry expression, but basal mCherry fluorescence was also different across the differentiated cells in UAS-BFP cells expressing no inducible system components (Fig. S4D). The Ert2-expressing line showed higher basal BFP, but only when differentiated into XEN-like cells, whereas no other transgene expression or differentiation affected baseline BFP signal. After induction, 4OHT and GZV were less efficacious at producing their payloads in Ecto and XEN cells, whereas ABA had no change in efficacy for Ecto cells but became significantly more efficacious in XEN cells. Finally, we also evaluated the effects of each system and each drug on the efficiency of the differentiation (Fig. S4E). Expression of the ABI/Pyl system diminished the fraction of Ecto cells produced during the differentiation and increased the fraction of XEN cells produced, but no other system affected differentiation efficiency simply through expression. Treatment with both ABA and GZV affected differentiation efficiency, with both systems suppressing the production of XEN cells.

These data suggest that our method of transgene insertion is broadly robust to silencing across common ESC differentiations, and that all systems continue to function as inducers in differentiated cells. However, each system showed unique properties in both its effects on the differentiation and in terms of the efficacy of its function in differentiated cells, suggesting that there is no easy formula for which system to use during differentiation; future users should determine empirically which system fits their model best.

### Juxtacrine-inducible systems drive BFP expression in mESCs

Juxtacrine signaling is an important mechanism in development and physiology that allows cells to identify their tissue context and coordinate their behavior (Kania and Klein, 2016; Siebel and Lendahl, 2017). Recognition of cell surface antigens has been exploited in cell engineering to modulate pathways and drive different cellular behaviors (Choe et al., 2021; Morsut et al., 2016; Roybal et al., 2016; Zhang et al., 2022). SynNotch and SNIPR are designed juxtacrine signaling receptors that have proven useful in cell engineering applications. SynNotch-dependent induction has been used successfully to control cell patterning and drive differentiation in multiple cell types (Garibyan et al., 2024; Lee et al., 2023; Malaguti et al., 2022). Previous SynNotch implementations in mESCs have shown robust activation and incredible potential for driving gene expression and differentiation (Malaguti et al., 2022). We sought to facilitate testing of these systems using rapidly iterable lentiviral techniques, previously used with hESCs (Lee et al., 2023), that could enable rapid prototyping of complex genetic circuits in mESCs. We further explored the efficacy of SNIPRs in mESCs, since in other cell types we found SNIPRs to produce both higher payload levels and to have more uniform activation across a transduced population (Weinberg et al., 2024).

**Fig. 3.**
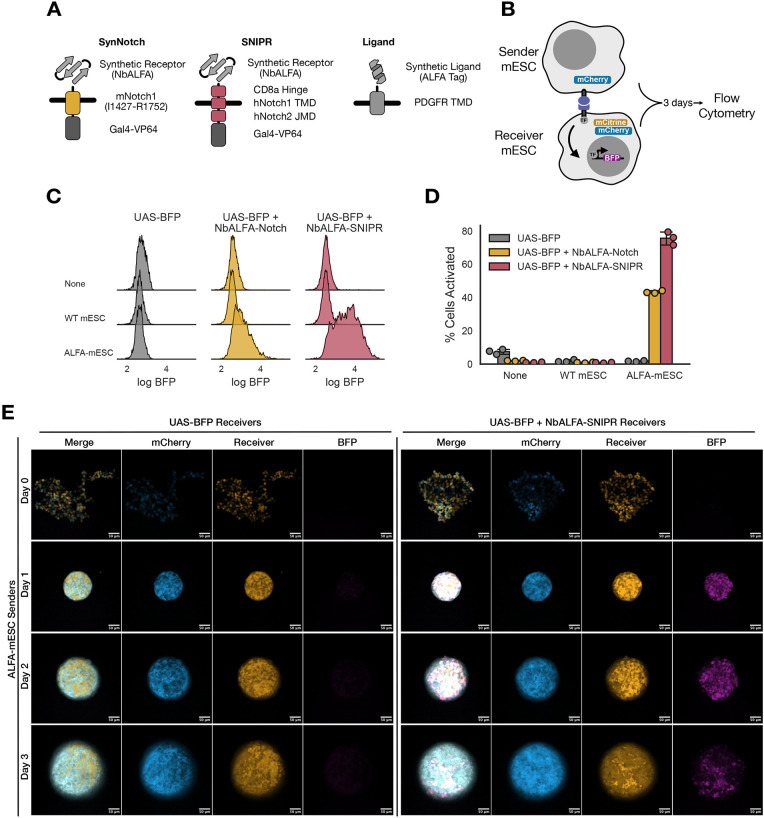
**Juxtacrine inducible systems activate BFP expression in mESCs.** (A) Schematic of components of synNotch, SNIPR and ligand. (B) Co-culture assay. (C) Histograms for population density estimates for BFP expression comparing UAS-BFP, UAS-BFP + NbALFA-Notch and UAS-BFP + NbALFA-SNIPR when cultured alone (None), with wild-type mESCs or with ALFA-mESCs for 3 days. (D) Comparison of the percentage of activated receiver mESCs (UAS-BFP, UAS-BFP + NbALFA-Notch and UAS-BFP + NbALFA-SNIPR) when cultured with the different sender mESCs (wild-type mESCs and ALFA-mESCs), as assessed by gating inactive cells at the 99th percentile of BFP expression in wild-type mESCs cultured alone. (E) Co-cultured ALFA-mESC sender cells with either UAS-BFP or UAS-BFP + NbALFA-SNIPR receiver cells in 2i at a 1:1 ratio in a low-attachment plate form spheres. Images taken every 24 h with confocal microscopy. Single slices from the middle of the *z*-stack are shown. Scale bars: 50 µm.

SynNotch and SNIPRs are both synthetic proteolytic receptors that drive transcription downstream of extracellular antigen recognition via customized sensing domains (Fig. 3A) (Morsut et al., 2016; Zhu et al., 2022). SNIPRs were developed by exploring the ability of a library of chimeric proteins to overcome the high background activity of synNotch in some cell types. Given our experience using SNIPRs in other heterologous systems (Kim et al., 2024; Weinberg et al., 2024), we suspected that SNIPRs would be better expressed, and have higher payload output and expression in mESCs, as we have seen in other cell types. We selected a SNIPR here that proved effective in recent studies in T cells (Kim et al., 2024) containing the human CD8a hinge domain, human Notch1 transmembrane domain and human Notch2 juxtamembrane domain.

We also sought to introduce a ligand-receptor recognition pair with high affinity and orthogonality from endogenous cell-surface proteins. Previous systems have used GFP or its non-fluorescent mutant nfGFP, which are orthogonal to endogenous proteins but can limit the available channels in fluorescence experiments while also presenting a minor challenge because of the size of the ligand (Fridy et al., 2014; Lee et al., 2023; Malaguti et al., 2022; Morsut et al., 2016; Toda et al., 2018, 2020). We sought to expand the available orthogonal ligands by using ALFA-tag as our ligand and its cognate binder NbALFA as our antigen-sensing domain (Götzke et al., 2019). We designed NbALFA as the extracellular recognition domain with either the original synNotch or SNIPR (Fig. 3A). For our ‘Receiver Cells’, cells that express a juxtacrine receptor and an output circuit capable of responding to receptor activation, we stably integrated synNotch or SNIPR using lentiviral transduction into the same output circuit with TagBFP in mESCs, as previously described (Fig. 3B). We sorted for similar mCherry expression and surface expression using Myc-tag for NbALFA-synNotch or NbALFA-SNIPR (Fig. S5A). SNIPR and SynNotch have modest transduction efficiency and expression, consistent with previous reports in other cell types (Kim et al., 2024). We designed the ALFA-tag ligand to be displayed on the PDGFR transmembrane domain with a fibcon rigid linker (Jacobs et al., 2012; Weinberg et al., 2024) (Fig. 3A) downstream of the Ef1a promoter with mCherry as a transduction marker using the MTK. We stably integrated this construct into mESCs by lentiviral transduction and sorted a polyclonal cell population using high mCherry expression and surface expression of ALFA-tag (Fig. S5B). This engineered mESC line (ALFA-mESC) represents the ‘Sender Cell’ (Fig. 3B) because it expresses the ligand that our receiver cells are capable of detecting.

To test for the functionality of our lentivirally generated cell lines, we co-cultured sender and receiver cells at a 1:1 ratio in a low-attachment plate. This culture method allowed homogeneous distribution of our cell types and forced them to grow into well-mixed spheres. We cultured wild-type mESCs without ligand (WT mESCs) alone as our control senders, ALFA-mESCs senders alone, output circuit mESCs (UAS-BFP) alone as our control receivers, and UAS-BFP + NbALFA-Notch or UAS-BFP + NbALFA-SNIPR receivers alone then set up co-cultures with each of the sender and receiver cells combined. Cultures were assessed after 3 days for BFP expression by flow cytometry (Fig. 3B). We saw that only in co-cultures of ALFA-mESCs with NbALFA-Notch or NbALFA-SNIPR on the receiver cell was there an increase in BFP expression. We saw a robust 1.5-2 log shift in BFP expression in UAS-BFP + NbALFA-SNIPR versus an approximately half log increase for UAS-BFP + NBALFA-Notch receiver cells (Fig. 3C), although both types of receiver cell showed a broad distribution of BFP expression. We quantitatively characterized activation by gating on the 99th percentile of BFP expression in mESCs cultured alone to separate activated cells from inactive cells in our cytometry histograms. When co-cultured with cells expressing UAS-BFP + NbALFA-SNIPR, the fraction of cells showing ALFA-tag-dependent BFP expression reached ∼80% while synNotch was slightly above 40% (Fig. 3D). We characterized the distribution of co-cultured sender and receiver cells using the UAS-BFP + NbALFA-SNIPR inducible system by confocal microscopy over 3 days (Fig. 3E, Fig. S5C). We see that wild-type mESCs (no reporter) or ALFA-mESC (mCherry) sender cells, and UAS-BFP mESCs (mCitrine) or UAS-BFP + NbALFA-SNIPR (mCitrine and mCherry) receiver cells form into a spheroid and are homogeneously distributed at day 1. Interestingly, by day 3, sender and receiver cells appear to segregate to opposite sides of the spheroid. This potentially may be due to the proliferative and colony-forming capacity of each cell relative to its location within the spheroid. Additionally, by day 3 we observe no necrosis within the middle of the spheroid. Consistent with our quantitative results, we see activation of BFP expression only in the co-cultures with ALFA-mESC sender cells and UAS-BFP + NbALFA-SNIPR receiver cells, and BFP expression is uniformly distributed throughout the spheroid even after senders and receivers have segregated at day 3. Altogether, these data indicate that both NbALFA-Notch and NbALFA-SNIPR juxtacrine inducible systems activate BFP expression in mESCs with NbALFA-SNIPR working with greater efficiency.

### Engineering GAL4-inducible mESCs to differentiate into neurons

**Fig. 4.**
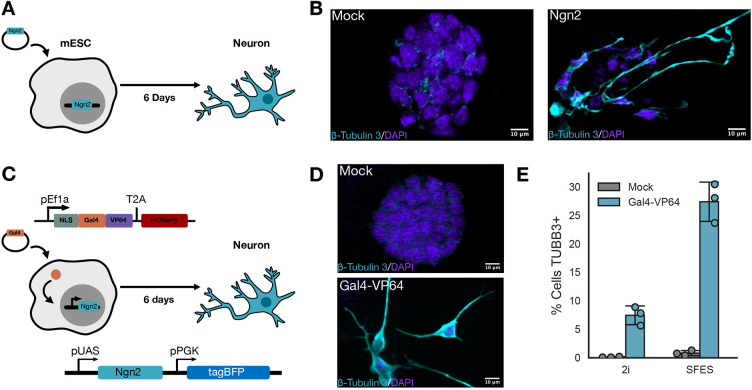
**Engineering Gal 4-inducible mESCs to differentiate into neurons.** (A) Schematic of the neuron differentiation program as previously described. (B) Neuronal validation using the class III β-tubulin (TUBB3) marker staining of cells cultured in 2i by confocal microscopy. Scale bars: 10 µm. (C) Schematic of the neuron differentiation program engineered in mESCs using the transcription factor Ngn2 downstream of the inducible UAS promoter. Gal4 transcription factor expressed on a lentiviral expression vector is transduced into an inducible Ngn2 mESC and results in expression of Ngn2. (D) Neuronal validation using TUBB3 staining of cells cultured in 2i by confocal microscopy. Scale bars: 10 µm. (E) The percentage of UAS-Ngn2 cells that differentiated in either mock-transduced or Gal4-VP64-transduced conditions grown in either 2i or SFES media, as assessed by gating on the 99th percentile of class III β-tubulin staining in mock-transduced mESCs cultured in 2i.

As an application of these systems in the context of cell differentiation, we first sought to engineer mESCs to differentiate into a population of a cell type of interest. Previous work has shown that ectopic expression of the Ngn2 transcription factor is sufficient to drive neuronal differentiation in mESCs (Thoma et al., 2012). We repeated these studies and verified the same results by transducing mESCs with a plasmid (Pfisterer et al., 2011) expressing Ngn2 downstream of the PGK promoter (Fig. 4A,B) and culturing the transduced cells in 2i for 6 days. Differentiation into neuronal cells was confirmed with the neuron-specific class III β tubulin (TUBB3) marker using immunofluorescence confocal microscopy.

We engineered mESCs with the Ngn2 transcription factor downstream of an inducible promoter. We expressed Ngn2 downstream of the same GAL4 UAS-ybTATA promoter used above (Fig. 4C). We stably integrated this cassette using lentivirus and selected based on constitutive expression of TagBFP as a marker of successful integration into mESCs, producing UAS-inducible neurons. We refer to these cells as UAS-Ngn2 mESCs. We designed a lentiviral expression vector that, when transduced into our UAS-Ngn2 mESCs, would express Gal4-VP64 to activate Ngn2 expression and cultured transduced cells for 6 days in 2i (Fig. 4C). We validated neuronal cells using the TUBB3 marker with immunofluorescence confocal microscopy (Fig. 4D). To determine the efficiency of differentiation in our UAS-Ngn2 mESCs and exclude the possibility of neurons arising due to random differentiation, we quantified the percentage of TUBB3-positive cells using flow cytometry in Gal4-VP64-transduced UAS-Ngn2 versus mock-transduced (viral packaging without Gal4-VP64 plasmid) UAS-Ngn2, as well as Gal4-VP64-transduced wild-type mESCs and mock-transduced wild-type mESCs. We cultured in both 2i and SFES media conditions, and compared the differentiation efficiency (Fig. 4E, Fig. S6). After Gal4-VP64 transduction, 30% of UAS-Ngn2 mESCs were class III β-tubulin positive (Fig. 4E, Fig. S6A,B). While previous work (Mulas et al., 2019) has shown that wild-type mESCs differentiate into neurons in SFES, our mock-transduced UAS-Ngn2 (Fig. 4E) and wild-type mESCs (Fig. S6C-E) did not differentiate. This may be due to multiple variations in the culture conditions used here versus the previously described protocol. Altogether, these data indicate that the GAL4 UAS-ybTATA promoter is capable of driving sufficient expression of Ngn2 to induce neuronal differentiation.

### Small molecule synthetic transcription factor systems induce neuronal differentiation in mESCs

**Fig. 5.**
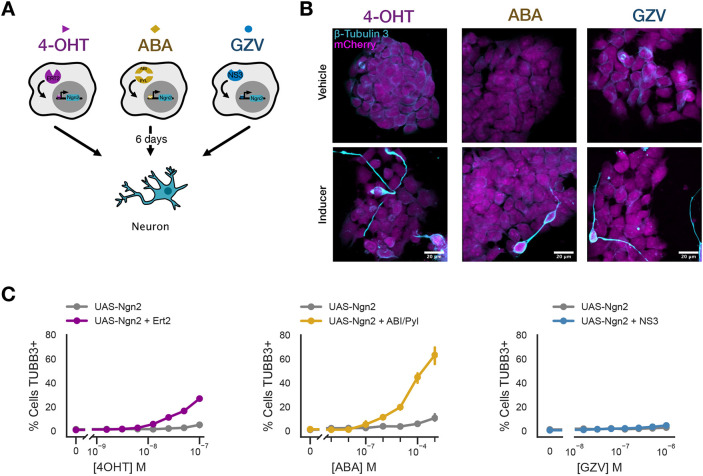
**Small molecule synthetic transcription factor systems induce neuronal differentiation in mESCs.** (A) Schematic of the assay used for small molecule synthetic transcription factor systems with Ngn2 downstream of the inducible system. (B) Small molecule-induced differentiation of mESCs into neurons cultured in 2i, using 50 nM of 4OHT, 625 nM of ABA and 62.5 nM of GZV. Validation was achieved by staining for the class III β-tubulin (TUBB3) marker using confocal microscopy. Maximum-intensity projections are shown. Scale bars: 20 µm. Images are not representative and were selected to identify any neurons seen on the coverslip. (C) The percentage of cells in each small molecule UAS-Ngn2 system that differentiated, as assessed by gating on the 99th percentile of TUBB3 staining in UAS-Ngn2 cells cultured in SFES without drug.

We next combined our UAS-Ngn2 mESCs with each of the small molecule-inducible systems. To test for differentiation, we stably integrated each synthetic transcription factor system into separate UAS-Ngn2 populations by lentiviral transduction (Fig. 5A). To validate differentiation, we cultured each system in 2i separately for 6 days at doses roughly equivalent to the EC_50_ for TagBFP in our initial testing of these systems, using 50 nM of 4OHT, 625 nM of ABA and 62.5 nM of GZV. We then validated neuronal differentiation with immunofluorescence confocal microscopy (Fig. 5B). We observed TUBB3-positive cells in all induced conditions, suggesting that the inducible systems we tested were capable of driving Ngn2-dependent differentiation.

Beyond the role of a master transcription factor, the quantitative requirements of direct differentiation via Ngn2 are poorly understood. We used the dose-dependent expression of our inducible systems to begin to explore the degree to which transcription factor expression level controlled differentiation efficiency. We repeated the above experiment using a range of doses for each inducible system while cultured in SFES. We quantified differentiation using immunofluorescence staining for TUBB3 with flow cytometry to enable high throughput evaluation of our various differentiation conditions (Fig. 5C, Fig. S7A). Both Ert2- and ABI/Pyl-inducible systems produced a dose-dependent increase in TUBB3 staining. We quantified differentiation by defining as differentiated all cells with TUBB3 staining greater than the 99th percentile of cells in the UAS-Ngn2-only line. Again, both Ert2- and ABI/Pyl- inducible systems produced a dose-dependent increase in differentiated cells compared to UAS-Ngn2 controls. Curiously, the NS3-inducible system was not able to efficiently differentiate at rates above background. We further validated and quantified neuronal cell fate specification for each system using immunofluorescence staining for Ngn2 and Sox2 with flow cytometry (Fig. S8A,B), and immunoblotting for MAP2 and Ngn2 (Fig. S8C). Consistent with staining for TUBB3, both Ngn2 and MAP2 were upregulated in Ert2- and ABI/Pyl-inducible systems in a dose-dependent manner. The relative induction of Ngn2 between the maximum doses of 4OHT and ABA, where ABA produced much more Ngn2, suggests that levels of Ngn2 expression are directly correlated to neuronal differentiation. In the NS3-inducible system, MAP2 expression was slightly upregulated above baseline while Ngn2 expression was only detectably different via immunofluorescence. Additionally, we see a downregulation of Sox2 in Ert2-and ABI/PYL-inducible systems, while it is sustained in NS3 at baseline, consistent with its previously characterized high expression levels in neural stem cell populations and downregulation as cells differentiate to post-mitotic neurons (Zhang and Cui, 2014). These results indicate that some but not all of these systems are capable of driving direct differentiation of mESCs into neurons, with important implications for the quantitative requirements of Ngn2-dependent directed differentiation**.**

### SNIPR induces neuronal differentiation in mESCs

**Fig. 6.**
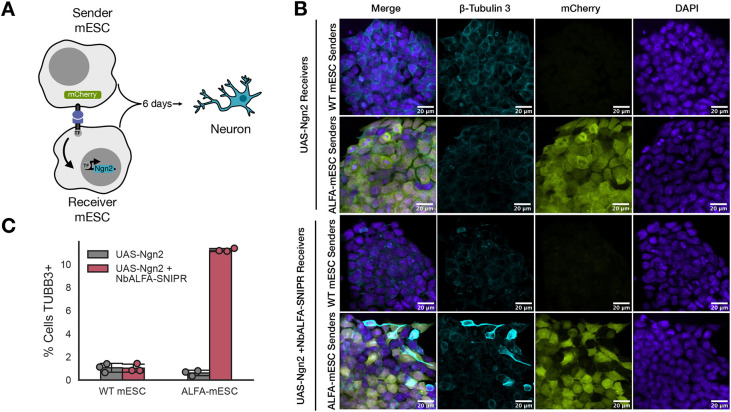
**SNIPR induces neuronal differentiation in mESCs.** (A) Schematic of a co-culture assay for ALFA-mESC sender cells and UAS-Ngn2 + NbALFA-SNIPR receiver cell with Ngn2 downstream of the inducible system (NbALFA-SNIPR UAS-Ngn2). (B) Validation of neuronal differentiation of mESCs co-cultured in 2i using the class III β-tubulin (TUBB3) as a marker with confocal microscopy. Maximum-intensity projections are shown. Scale bars: 20 µm. (C) Comparison of the percentage of receiver mESCs (UAS-Ngn2 and UAS-Ngn2 + NbALFA-SNIPR) when cultured in SFES with the different sender mESCs (wild-type mESCs and ALFA-mESCs) that differentiated, as assessed by gating on the 99th percentile of TUBB3 staining in wild-type mESCs cultured alone in SFES.

We tested differentiation downstream of juxtacrine induction. Differentiation using synNotch has previously been described (Garibyan et al., 2024; Lee et al., 2023; Malaguti et al., 2022), so we tested for differentiation using SNIPR, which showed higher expression and percentage of cells expressing the payload of the receptor in our hands. We stably integrated NbALFA-SNIPR into our UAS-Ngn2 cells by lentiviral transduction, as described above. We then cultured wild-type mESCs (no ligand) alone as our control senders, ALFA-mESCs senders alone, output circuit mESCs (UAS-Ngn2) alone as our control receivers, UAS-Ngn2 + NbALFA-SNIPR receivers alone and set up co-cultures with each of the sender and receiver cells combined. We validated differentiation after 6 days of culture in 2i by confocal microscopy and quantified differentiation after 6 days of culture in SFES by flow cytometry (Fig. 6A-C, Fig. S9A). We saw differentiation only in co-cultures of UAS-Ngn2 + NbALFA-SNIPR receivers and ALFA-mESC senders. Furthermore, in our microscopy assay we observed spatially dependent patterning where, qualitatively, receivers adjacent to senders seemed more likely to differentiate (Fig. 6B). These results indicate that SNIPR is capable of driving direct differentiation of mESCs into neurons in a cell contact-dependent and spatially dependent manner.

## DISCUSSION

In this work, we have demonstrated drug- and cell contact-inducible systems for controlling expression and differentiation in mESCs. We show that three small molecule-inducible transcription factor systems as well as two juxtacrine inducible systems work reliably and efficiently in mESCs, as measured by expression of BFP output. We demonstrate the functionality of the GAL4 UAS-ybTATA inducible promoter as a transcriptional regulator in mESCs. Additionally, we show that, when applied in the context of differentiation, some of these inducible systems can drive direct differentiation of mESCs into neurons by driving expression of the transcription factor Ngn2. Through this work, we expand the toolkit for engineering mESCs and provide a rapid, robust and iterable model for engineering mESCs. The tools we present enable quantitative investigation of the requirements for differentiation, and offer multi-dimensional control over development in multiple modalities. Each of the components demonstrated in our study can theoretically be used on their own or in combination, in applications that aim to further our understanding of developmental principles.

We characterized various aspects of our small molecule-inducible systems in mESCs to help inform future users of these systems. While we cannot make direct comparisons with the original synZiFTR system (Li et al., 2022), it is interesting that the performance of the output payload expression of each of our small molecule systems in mESCs differs considerably from the synZiFTR system used in Jurkat and primary T cells. Specifically, in our systems, ABA shows the highest payload expression followed by 4OHT and GZV when used in mESCs. In the synZiFTR system, GZV shows highest payload expression followed by 4OHT and ABA in Jurkat T cells. While GZV shows less robust expression in mESCs, it is worth noting that such a system is useful in cases where minimal activation is necessary and sufficient for controlling amplification of signaling events in a developmental context, e.g. in driving a morphogen cascade. Additionally, the variation in performance we see for each system across differentiation states further highlights the importance of determining the best system to use, depending on application- and cell type-specific contexts. Although not all of the small molecule systems affect differentiation, we do observe effects on differentiation that either change differentiation potential or change the kinetics of differentiation. Among the advantages of using any of these inducible systems is their theoretical orthogonality to existing systems used in the study of development. This includes the widely used TET system (Gossen and Bujard, 1992; Gossen et al., 1995; Pedone et al., 2019). Our small molecule-inducible systems complement TET inducible expression while yielding, in some cases, a higher payload output (Fig. S10). We think our GAL4-dependent systems are complementary to the existing TET and synZiFTR systems.

We show that we can achieve cell contact-inducible gene expression in mESCs using synNotch and with even greater efficiency by using SNIPR. We achieved this using lentiviral integration and sorting for the highest expressing polyclonal population, an approach that took only over a week to generate a new cell line, and is therefore amenable to rapid iteration when prototyping gene circuits. We used ALFA-tag antigen on sender cells with the NbALFA nanobody on receiver cells to further expand the possibilities for orthogonal juxtacrine signaling. In the previously described SyNPL system (Malaguti et al., 2022), this was achieved through single-site integration of the output circuit followed by clonal selection and subsequent random genetic insertion of SynNotch, and again followed by clonal selection. Isolation of the most efficient clone was achieved through the use of EGFP antigen on sender cells with the anti-GFP nanobody (LaG17) on the receiver cells. While the SyNPL system shows greater activation of gene expression (>90% for SyNPL compared to >80% for the SNIPR used in this study), it comes at the expense of the potential for rapid prototyping of complex genetic circuits in mESCs. Given the advantages of each of these systems and the orthogonality of the ligands used, it is up to the user to determine the best system and which modules of complementation to use in their experimental design. We propose that a happy medium between these approaches, i.e. iterating using lentiviral approaches and then generating a final cell line for use via clonal selection, may be the optimal approach for most users.

Successfully engineering cellular behavior requires considerable iteration, refinement and optimization. Enabling rapid cycles of cell engineering makes mESCs a more accessible model for this work. Our ability to study and understand developmental principles could greatly benefit from tools that expedite the process of asking developmental questions. The lentiviral transduction system we report here, which is compatible with the previously published Mammalian Toolkit for rapidly generating genetic components for cell engineering, enables rapid, robus and iterable engineering workflows in mESCs. This system complements high-efficiency landing pads (Chow et al., 2021; Fonseca et al., 2019; Matreyek et al., 2020) in cases where controlling copy number is essential.

The different abilities of inducible systems to induce differentiation begs questions about quantitative requirements for differentiation. For example, when differentiating our UAS-Ngn2 lines into neurons using the NS3 system, we do not see differentiation as measured by TUBB3 staining, as seen in the Ert2 and ABA/Pyl systems. However, we begin to see Ngn2 expression at increasing GZV concentration (Fig. S8). This suggests that a necessary amount of pro-differentiation transcription factor is needed to be in the ‘ON’ state for differentiation to occur, which is otherwise unmet by the NS3 system at the GZV levels we tested. How do the kinetics of transcription factor production contribute to differentiation? We think that the quantitative data we show where we couple Ngn2 expression (via both immunofluorescence and immunoblotting) to differentiation, as indicated by TUBB3 expression and MAP2 expression indicates the required Ngn2 expression regime for inducing differentiation.

Through this study, we present additional tools that expand the synthetic developmental biology toolkit. Because of the complexity of development, synthetic development requires multi-dimensional control over multiple scales necessary to understand and build more complex developmental systems. The tools presented here add to a growing toolkit that enables this multidimensional multiscale control over cellular behavior. Although we show the efficacy of these tools in a relatively simple expression system to direct mESC into neurons, these tools are ready to be integrated into more-complex synthetic circuitry, such as feedback controllers (Milias-Argeitis et al., 2011; Vecchio et al., 2017). Additionally, implementing these systems into building more controlled and refined *in vitro* culture models, such as organoids, by controlling cell fate, timing of differentiation and organization, will give us a deeper understanding of tissue composition and ultimately of predictive tissue engineering (Trentesaux et al., 2023).

## MATERIALS AND METHODS

### Cell culture

ES-E14TG2a (ATCC CRL-1821) mESCs acquired from ATCC without further testing or authentication were cultured in serum-free ES media (SFES) supplemented with 2i and LIF as described by Mulas et al. (2019). SFES media consists of 500 ml DMEM/F12 (ThermoFisher Scientific, 21331020), 500 ml Neurobasal (Gibco #21103-049), 5 ml N2 Supplement (Gibco #17502-048), 10 ml B27 (ThermoFisher Scientific, 17504044), 6.66 ml 7.5% BSA (Gibco #15260-037), 10 ml 100× GlutaMax (Gibco #35050-061), 1 ml 55 mM 2-mercaptoethanol and 10 ml of Antibiotic-Antimycotic. To make ‘2i’ medium, 1 μM PD03259010 (Selleckchem, S1036), 3 μM CHIR99021 (Selleckchem, S2924) and 10^4^ units/ml LIF (ESGRO, ESG1107) were added to 45 ml SFES. To passage, mESCs were treated with 0.5 ml of accutase in a six-well plate (Corning, 353046) for 5 min at room temperature. After incubation, cells were mixed by pipette and moved to a 15 ml conical tube, supplemented with 10 ml wash medium and spun at 400 ***g*** for 4 min. Wash medium consists of DMEM/F12 (ThermoFisher Scientific, 21331020) supplemented with 8 ml 7.5% BSA. Wash medium was then removed and cells were counted using the Countess II Cell Counter (ThermoFisher Scientific) according to the manufacturer's instructions. Cells were then plated in six-well plates that had gelatinized with 1% gelatin for 30 min at 37°C at 1.5×10^5^ cells per well in 2 ml of 2i. Cells were split every other day or every 3 days (10^5^ cells). All cells were maintained at 37°C with 5% CO_2_.

Lenti-X 293T packaging cells (Clontech, 11131D) were cultured in medium consisting of Dulbecco's Modified Eagle Medium (DMEM) (Gibco, 10569-010) and 10% fetal bovine serum (FBS) (University of California, San Francisco Cell Culture Facility). Lenti-X 293T cells were cultured in T150 or T225 flasks (Corning, 430825 and 431082), and passaged upon reaching 80% confluency. To passage, cells were treated with TrypLE express (Gibco, 12605010) at 37°C for 5 min. 10 ml of medium was then used to quench the reaction and cells were collected into a 50 ml conical tube and pelleted by centrifugation (400 ***g*** for 4 min). Cells were cultured until passage 30, whereupon fresh Lenti-X 293T cells were thawed. All cells were maintained at 37°C with 5% CO_2_.

### DNA constructs

The output circuit, pHR_Gal4UASpyb–TATA_tBFP_pGK_mCitrine and the SNIPR plasmid were generous gifts from Dr Kole Roybal (UCSF, CA, USA). The small molecule synthetic transcription factors, the sender and receiver juxtacrine plasmids, and the TET system were constructed using the Mammalian Toolkit (MTK) (Fonseca et al., 2019), a hierarchical DNA assembly method based on Golden-Gate (GG) cloning (Engler et al., 2009). The SNIPR used in this study contains the CD8a hinge domain, the human Notch1 transmembrane domain and the human Notch2 juxtamembrane domain. The SNIPR was domesticated as an MTK part 3B with the Gal4-VP64 transcriptional actuator domain. The juxtacrine sender cell construct was assembled using the MTK. The ALFA epitope tag (Götzke et al., 2019) and a single fibcon domain (Jacobs et al., 2012) was generated as part 3A. The PDGFRb transmembrane domain was generated as part 3B. The receiver cell plasmid consisted of the standard SNIPR core with an anti-ALFA tag nanobody as a binder. All constructs were assembled into a novel lentiviral destination backbone (Kim et al., 2024; Weinberg et al., 2024) via a PaqCI reaction following the manufacturer's instructions (New England Biolabs, R0745S). The Ngn2 plasmid was Addgene plasmid #34999 (deposited by Malin Parmar, Lund University, Sweden). The UAS-NGN2 plasmid was constructed using Gibson Assembly with In-Fusion^®^ Snap Assembly Master Mix (Takara Bio, 638947) and following recommended manufacturer instructions. The TRE-BFP and TRE-Ngn2 plasmids were constructed using NEBuilder HiFi DNA Assembly Master Mix (E2621S) and following recommended manufacturer's instructions. All plasmids were propagated in Stbl3 *E. coli* (QB3 MacroLab). Plasmids were verified via sequencing or restriction digest. All plasmids have been deposited in Addgene (deposit IDs listed in Table S1). Details for key constructs used in this study are provided in Fig. S1 and Table S1. All construct sequences are available on Zenodo (Soliman et al., 2024).

### Lentiviral transduction and generation of stable cell lines

Lenti-X 293T cells (Takara Bio, 632180) were seeded at ∼7×10^5^ cells/well in a 6-well plate to yield ∼80% confluency the following day. The following day cells were transfected with 1.5 μg of transfer vector containing the desired expression cassette, and the lentiviral packaging plasmids pMD2.G (170 ng) and pCMV-dR8.91 (1.33 μg) using 10 μl of Fugene HD (Promega, E2312), according to manufacturer procedure. At 48 h the viral supernatant was filtered through a 0.45 μm PVDF filter and concentrated using the Lenti-X concentrator (Takara, 631231) according to the manufacturer's instructions. Lenti-X concentrator solution was added at a 1:3 viral supernatant:concentrator ratio, mixed by inversion and incubated at 4°C for at least 2 h. Supernatant-concentrator mix was pelleted by centrifugation at 1500 ***g*** at 4°C for 45 min, supernatant was removed and pellet was resuspended using 100 μl 2i medium for each T25 flask of mESCs. Two wells of a 6-well plate were concentrated and applied to one T25 flask plated with 500,000 cells on the day of transduction. 72 h after the addition of the viral supernatant to mESC culture, polyclonal cell populations were selected via fluorescence-activated cell sorting (FACS), as described below. Cells were sorted using BD FACS Aria sorter.

### Small molecule-inducible system assays for BFP

Stock solutions of abscisic acid (Sigma Aldrich, 862169, 50 mM in ethanol), 4-hydroxytamoxifen (Sigma Aldrich, H6278, 1 mM in ethanol) and grazoprevir (MedChemExpress, HY-15298, 1 mM in DMSO) were stored at −80°C. 1×10^4^ mESCs were seeded into 96-well gelatin-coated flat-bottomed plates in 100 μl 2i medium on day 0 of induction. On day 1 of induction,100 μl of medium containing concentrated amounts of 2× 4-OHT, 10× ABA and 3× GZV was added to maximum dosage row of wells and serial dilution was performed across each row, ending on diluent-only in the minimum dosage row. On day 4, cells were lifted and transferred to 96-well round-bottomed plates and prepared for flow cytometry as described below. All inductions were performed in biological triplicates.

### Small molecule-inducible system differentiation assays for BFP

For differentiation into ectoderm-like and XEN-like cells, small molecule-inducible mESC lines were seeded at 2×10^3^ cells per well in 96-well gelatin-coated flat-bottomed plates in 100 μl SFES (-2i, -LIF) medium with 0.25 μM retinoic acid (RA) on day 0 of induction. 24 h after plating, 100 μl of SFES+RA medium containing the maximum dose of each drug (100 nM 4OHT, 1 mM ABA or 1 µM GZV) for the treated condition or containing diluent for the untreated condition was added to respective inducible system lines. 96 h after plating, cells were stained with UV live/dead stain dye, then lifted and transferred to 96-well round-bottomed plates. Cells were live stained with identity surface markers and prepared for flow cytometry as described below. For differentiation into epiblast-like cells, small molecule-inducible mESC lines were seeded at 5×10^4^ cells per well in 96-well gelatin-coated flat-bottomed plates in 200 μl SFES containing the maximum dose of each drug for the treated condition or containing diluent for the untreated condition was added to respective inducible system lines. 72 h after plating, cells were stained with UV live/dead stain dye, then lifted and transferred to 96-well round-bottomed plates. Cells were prepared for intracellular staining and flow cytometry, as described below. All inductions were performed in biological triplicates.

### Small molecule effects on pluripotency assays

Wild-type mESCs were seeded at 1×10^4^ cells per well in 96-well gelatin-coated flat-bottomed plates in 100 μl of 2i. The next day, 100 μl of 2i or SFES with maximum dose of each drug (100 nM 4OHT, 1 mM ABA or 1 µM GZV) or with diluent was added. Additionally, wild-type mESCs were seeded at 2×10^3^ cells per well in 96-well gelatin-coated flat-bottomed plates in 100 μl of 2i or SFES with 0.25 μM retinoic acid (RA). The next day, 100 μl of 2i or SFES with 0.25 μM retinoic acid (RA) and maximum dose of each drug (100 nM 4OHT, 1 mM ABA or 1 µM GZV) or with diluent was added. After 4 days in culture, cells were prepared for intracellular staining for pluripotency markers and assayed by flow cytometry, as described below. All inductions were performed in biological triplicates.

### Small molecule-inducible system comparing gene expression for BFP

Small molecule-inducible mESCs were generated as described above. Cells were sorted for all mCherry-positive cells generating 1.5 log range in expression in comparison to originally high sorted lines. Original and new cell lines for each small molecule system were seeded at 1×10^4^ in 2i with varying drug concentrations, as described above. Cells were assayed for BFP expression by flow cytometry 24 h after adding drug, as described below. Additionally, original and new cell lines were assayed for BFP expression by flow cytometry 72 h after treatment with drug (100 nM 4OHT, 1 mM ABA or 1 µM GZV) or with diluent, as described below. All inductions were performed in biological triplicates.

### Small molecule-inducible system assays for Ngn2

500 mESCs were seeded into 96-well gelatin-coated flat-bottom plates in 100 μl 2i medium on day 0 of induction. On day 1 of induction, 2i medium was exchanged for 100 μl of SFES medium. 100 μl of SFES containing 2× 4-OHT, 10× ABA and 3× GZV was added to maximum dosage row of wells and serial dilution was performed across each row, ending with diluent-only on the minimum dosage row. On Day 4, medium was refreshed with fresh induction medium. On day 6, cells were prepared for intracellular staining and flow cytometry, as described below. All inductions were performed in biological triplicates.

### Small molecule-inducible systems comparison to TET systems for BFP and Ngn2

TRE-BFP cell lines were generated as described above. Cells were seeded at 1×10^4^ cells per well in 96-well gelatin-coated flat-bottomed plates in 100 μl of 2i. The next day, 100 μl of 2i with maximum dose of each drug (100 nM 4OHT, 1 mM ABA, 1 µM GZV, or 1 µM doxycycline) or with diluent was added. Cells were assayed 72 h after adding drug for BFP expression by flow cytometry, as described below. For TRE-Ngn2, cell lines were generated as described above. Cells were seeded at 500 cells per well in 96-well gelatin-coated flat-bottomed plates in 100 μl of 2i. The next day, medium was exchanged for 200 μl of SFES containing the maximum dose of each drug (100 nM 4OHT, 1 mM ABA, 1 µM GZV or 1 µM doxycycline) or containing diluent was added. After 6 days, cells were prepared for intracellular staining and flow cytometry, as described below. All inductions were performed in biological triplicates.

### Antibody staining live cells

All experiments using antibody staining were performed in 96-well round-bottomed plates. After dissociation, cells for these assays were pelleted by centrifugation (400 ***g*** for 4 min) and supernatant was removed. Cells were resuspended for 45 min with appropriate antibodies in a staining solution of 25 μl PBS. After incubation, plates were spun down, rinsed with DPBS and resuspended in flow buffer made of DPBS with 2% FBS and 2 mM EDTA (ThermoFisher Scientific, AM9260G).

### Antibodies

Antibodies used for live cell flow cytometry assays include Alexa Fluor 647-Anti-Myc tag-conjugated antibody (Cell Signaling Technologies, 2233S) used at 1:100, FluoTag-X2 anti-ALFA Atto-488-conjugated antibody used at 1:50, Cd24 rat anti-mouse APC, Clone: M1/69 (BD Biosciences, 562349) used at 1:100, and Bv786 rat anti-mouse CD140a Clone APA5 (BD Biosciences, 740930) used at 1:100. All antibodies were diluted in DPBS (UCSF Cell Culture Facility) for staining. For FACS, all antibodies were used at 1:50 in 200 μl.

Antibodies for fixed intracellular staining assays include FluoTag-X2 anti-ALFA Atto-488 (NanoTag Biotechnologies, N1502-At488-L)-conjugated antibody diluted at 1:50, anti-beta III tubulin (EP1569Y)-Alexa Fluor 647 (Abcam, ab190575)-conjugated antibody used at 1:500, FluoTag-X2 anti-TagFP-Alexa 647 (NanoTag Biotechnologies,N0502-AF647-L)-conjugated antibody diluted at 1:500, and Nanog (D2A3) XP(R) Rabbit mAb PE conjugated antibody (Cell Signaling Technology, 60356S) diluted at 1:50. Primary antibodies include rat anti-Sox2 (ThermoFisher Scientific, 14-9811-82) diluted at 1:500, mouse anti-Oct3/4 (BD Biosciences, 611203) diluted at 1:500, ms-anti-beta III Tubulin (2G10) (Abcam, ab78078) diluted at 1:500 and rabbit anti-Neurogenin 2 (ThermoFisher Scientific, PA5-78556) diluted at 1:100. Secondary antibodies were diluted at 1:1000 and include anti-rabbit Alexa Fluor 488 (ThermoFisher Scientific, A11008), anti-mouse Alexa Fluor 488 (ThermoFisher Scientific, A-11001), anti-mouse Alexa Fluor 568 (ThermoFisher Scientific, A-11004), anti-rabbit Alexa Fluor 647 (ThermoFisher Scientific, A-21244), anti-rat Alexa Fluor 647 (ThermoFisher Scientific, A-21247) and anti-mouse Alexa Fluor 647 (ThermoFisher Scientific, A-21235). All antibodies were diluted in blocking buffer as described in the Bioscience Foxp3/Transcription Factor Staining Buffer Set (ThermoFisher Scientific, 00-5523-00), except when assessing Ngn2 expression, as described below.

Antibodies used for immunofluorescence include anti-beta III Tubulin antibody (EP1569Y) (Abcam, ab52623) used at 1:500 dilution and anti-rabbit Alexa Fluor 647 secondary antibody (ThermoFisher Scientific, A-21244) used at 1:2000. All antibodies were diluted in blocking buffer.

Antibodies for immunoblot include rabbit anti-Neurogenin 2 (Cell Signaling Technology, 13144S, 1:1000 dilution), anti-MAP2 (Abcam, ab32454, 1:200 dilution), HRP-conjugated GAPDH (Proteintech, HRP-60004, 1:5000 dilution) and goat anti-rabbit HRP-conjugated secondary (Bio-Rad, 1706515, 1:2000 dilution).

### FACS

Cell lines were bulk sorted for high expression using the UCSF Laboratory for Cell Analysis Core Facility FACSAriaII (BD Biosciences). Cells were assessed for BFP (405 nm excitation, 525/50 nm emission and 505lp collection dichroic), mCitrine (488 nm ex, 530/30 nm em and 505lp cd), mCherry (561 nm ex, 610/20 nm em and 600lp cd) and Alexa647 (633 nm ex and 670/30 nm em) fluorescence. Fluorescence-negative controls were used to set detector power so that negative cells appeared to have mean fluorescence ∼100 counts, and then transduced cells were sorted for cells with expression outside the negative control expression level. All data were collected using FACSDiva (BD Biosciences).

### Flow cytometry

All flow cytometry data was obtained using a LSR Fortessa or LSRII (BD Biosciences). All assays were run in a 96-well round-bottomed plate (ThermoFisher Scientific, 08-772-2C). Samples were prepared by pelleting cells in the plate using centrifugation at 400 ***g*** for 4 min. Supernatant was then removed and 200 μl of PBS (UCSF Cell Culture facility) was used to wash cells. The cells were pelleted again, as described above, and supernatant was removed. Cells were resuspended in 200 μl of flow buffer (DPBS with 2% FBS and 2 mM EDTA) and mixed by pipetting prior to flow cytometry assay. At least 1×10^7^ events were recorded for all experiments.

### synNotch and SNIPR activation assays

mESCs expressing synNotch and SNIPR constructs were seeded at a density of 5×10^4^ cells/well in a low-attachment round-bottomed 96-well plate (Corning, 7007). Cells were plated alone or with an equal number of either wild-type mESCs or mESCs expressing the ALFA-tag ligand in a total of 200 μl of 2i medium. Plates were spun briefly (400 ***g*** for 1 min) to increase likelihood of cell–cell interaction. Cells were co-cultured for 72 h and the BFP expression and surface expression of receptors and ALFA-tag were measured using flow cytometry. synNotch and SNIPR activation was by gating on the 99th percentile of BFP expression in the negative control, with all cells with higher BFP expression considered ‘on’.

For imaging the SNIPR co-culture assays, cells were plated as described above at a density of 300 cells/well alone or at a 1:1 ratio and imaged on day 0. Wells were imaged every 24 h for 3 days. Images were captured by confocal microscopy using the Opera Phenix automated spinning-disk confocal microscope with a 10× air objective in 96-well plates. Single slice images from the middle of the *z*-stack were exported from the manufacturer's software (Harmony) and processed on Fiji (Schindelin et al., 2012). All co-culture inductions were performed in biological triplicates.

### SNIPR activation-Ngn2 differentiation assay

mESCs expressing SNIPR constructs were seeded at a density of 10^4^ cells/well in a low-attachment round-bottomed 96-well plate. Cells were plated alone or at 1:1 ratios with mESCs expressing the ALFA-tag ligand in a total of 200 μl of 2i medium. Plates were spun briefly (400 ***g*** for 1 min) to increase likelihood of cell-cell interaction. The next day, 2i medium was exchanged for SFES. Cells were co-cultured for 6 days then fixed as described above. β- III tubulin expression and surface expression of receptors and ALFA-tag were measured using flow cytometry. SNIPR activation and differentiation were assessed by gating on the 99th percentile of class III β-tubulin staining in the negative control population of each experiment.

### Ngn2-dependent direct differentiation

Gal4-Ngn2, UAS-Ngn2 or wild-type cells were seeded at a density of 100,000 in a six-well plate of 2i medium the day before viral infection. On the day of transduction, one well of a six-well plate of virus containing the Gal4 plasmid was concentrated as described above and added to one well of Ngn2 and wild-type cells. Mock-transduced UAS-Ngn2 and wild-type cells underwent the same procedure. Cells were incubated for 48 h then polyclonal populations were sorted for expression of TagBFP and mCherry. Cells were then plated in gelatin-coated 96-well flat-bottomed plates at 10^3^ cells/well in 2i medium. The following day, medium was refreshed in cells under 2i medium conditions and exchanged with SFES in the case of SFES medium conditions. Cells were incubated for 14 days with medium refreshed every 2 days. Cells were then fixed and analyzed by flow cytometry, as described above.

### Immunofluorescence and fixed cell microscopy

Glass coverslips (ThermoFisher Scientific, 12-545-81P) were coated with 5 μg/ml fibronectin (Sigma-Aldrich, F0895-1MG) in HBSS (ThermoFisher Scientific, 14-025-092) and placed in 24-well plates (Fisherbrand #FB012929). Cells were lifted from 96-well plates and placed into 2i medium and cultured for an additional 3 days. Cells were fixed in 4% PFA/PBS for 20 min. Blocking and permeabilization were performed in a blocking solution consisting of 0.2 M glycine, 2.5% FBS, 0.1% Triton X-100 in 1×PBS for 30 min at room temperature. Primary and secondary antibodies were diluted and incubated in the blocking solution. Cells were incubated with primary antibodies for 30 min at room temperature and washed three times with PBS. Cells were then incubated with secondary antibodies for 30 min at room temperature and washed three times with PBS. Coverslips were then mounted in ProLong DAPI Fluoromount (ThermoFisher Scientific, P36966) onto the slide. Images were collected using a Nikon Ti inverted microscope equipped with Yokogawa CSU-22 spinning disk confocal and a custom 4-line solid state laser launch (100 mW at 405, 488, 561 and 640 nm excitation) using 40× air objective and processed using Fiji (Schindelin et al., 2012).

### Fixed cell staining to assess differentiation

Medium was removed from cells in 96-well flat-bottomed plates. Cells were treated with a Zombie UV Viability Kit (Biolegend, 280.423107) diluted 1:500 in PBS for 30 min in the dark at room temperature. Cells were lifted with accutase/mESC wash and transferred to a 96-well round-bottomed plate. Cells were pelleted at 400 ***g*** for 4 min and supernatant was removed. Cells then underwent fixation and permeabilization using an Biosciences Foxp3/Transcription Factor Staining Buffer Set (ThermoFisher Scientific, 00-5523-00) following the manufacturer's recommended procedure. After the fixation/permeabilization process, cells were stained for 1 h at room temperature in the dark. The stain was then washed off with the perm/wash buffer in the Biosciences kit twice and resuspended in flow buffer. Cells were analyzed by flow cytometry.

For assessing Ngn2 expression, cells were treated as described above up to removal of mESC wash supernatant. Cells underwent fixation in 96-well round-bottomed plates using 200 μl of 1.6% PFA in PBS for 10 min at room temperature and protected from light. Cells were pelleted at 600 ***g*** for 4 min and supernatant was removed. Cells were washed in 200 μl of 10% FBS in PBS. Cells were pelleted at 600 ***g*** for 4 min and supernatant was removed. Wash, centrifuge and supernatant removal steps were carried out twice. Cells were resuspended in 150 μl of ice-cold 90% methanol in PBS, incubated at 4°C for 1 h and protected from light. 100 μl of 10% FBS in PBS was then added to cells. Cells were pelleted at 600 ***g*** for 4 min and supernatant was removed. Wash, centrifuge and supernatant removal steps were carried out twice. Cells were stained in 25 μl of primary antibodies diluted in 2% FBS in PBS overnight at 4°C and protected from light. On day 2, cells were washed twice with 10% FBS in PBS as described above. Cells were stained in 25 μl of secondary antibodies diluted in 2% FBS in PBS at room temperature for 1 h and protected from light. Cells were washed twice as described above. Cells were resuspended in flow buffer and analyzed by flow cytometry.

### Immunoblotting

Cells were seeded in a 12-well plate at a density of 5.5×10^3^ cells/well for each condition. For 4OHT and ABA maximum concentration conditions, three additional wells were seeded and pooled for lysis. Cells were induced to differentiate as described above at 0 (ethanol, ethanol or DMSO), middle (25 nM 4OHT, 10 µM ABA or 111 nM GZV) and maximum (100 nM 4OHT, 1 mM ABA or 1 µM GZV) concentrations. After 6 days of differentiation, cells were lysed directly in the plate in Laemmli sample buffer (Bio-Rad) supplemented with 10% 2-betamercaptoethanol (Bio-Rad) in a volume of buffer estimated to result in equal protein concentrations across all wells. Samples were separated by gel electrophoresis on a 4-15% gradient SDS-PAGE gel and then transferred to a nitrocellulose membrane. Membranes were blocked in 5% milk in TBST for 1 h and then incubated with primary antibody in milk block overnight. Membranes were washed three times with TSBT, then incubated with HRP-conjugated secondary antibody for 1 h. After three washes with TBST and two washes with TBS, membranes were developed in SuperSignal Femto chemiluminescent substrate (ThermoFisher Scientific) and imaged on a ChemiDoc chemiluminescence imaging system (Bio-Rad). To probe for loading control, membranes were stripped for 15 min in Restore Stripping Buffer (ThermoFisher Scientific) and then re-blocked and incubated with primary antibody for 1 h before being developed as described above. All immunoblot images presented here were adjusted for contrast in Fiji (Schindelin et al., 2012) and then the contrast adjusted image was copied directly into the final figure, with minor stretching adjustments to line up lanes from different parts of the blot.

### Data presentation, analysis and availability

All experiments were performed in at least biological triplicates unless otherwise noted. Central tendency for individual replicates are presented as circles where possible. For BFP expression experiments, raw fluorescence is reported as median fluorescence value for all cells within a single replicate, and the presented means and errors are calculated between replicates. For class III β-tubulin staining experiments, raw fluorescence is reported as the mean fluorescence value for all cells within a single replicate, and the presented means and errors are calculated between replicates. For live cell flow cytometry, collected events were filtered to remove small events and then gated on FSC and SSC to capture singlet populations. For fixed-cell flow cytometry, small events were filtered out. Histogram density estimates represent all events for all three replicates in a flow cytometry experiment, and are calculated via the kdeplot function in seaborn with bandwidth adjustment of 0.2. All data analysis was conducted using custom Python scripts, available in GitHub (Soliman and Weinberg, 2024). Analysis was conducted in Jupyter (Kluyver et al., 2016) and relied on numpy (Harris et al., 2020), matplotlib (Caswell et al., 2023; Hunter, 2007), seaborn (Waskom, 2021), pandas (McKinney, 2010; https://github.com/pandas-dev/pandas), SciPy (Virtanen et al., 2020), scikit-learn (Pedregosa et al., 2011) and fcsparser. In cases where effect sizes were large and obvious, no statistical comparison was conducted. In all cases where statistics are displayed, they were calculated as a paired two-sample Student's *t*-test between the indicated samples. ALFA tag and NbALFA schematics were inspired by their published structures and presented using elements from a proposed protein emoticon (https://github.com/whitead/protein-emoji). All primary data from flow cytometry experiments and immunofluorescence microscopy are available on Zenodo (Soliman et al., 2024). All figures were assembled using Affinity Designer 2.

## Supplementary Material



10.1242/develop.204505_sup1 Supplementary information

Table S1. DNA constructs
