# Supplementary material for: Small molecule- and cell contact-inducible systems for controlling expression and differentiation in mouse embryonic stem cells
Source: Development. 2025 Jun 10;152(11):dev204505. doi: 10.1242/dev.204505 (PMC12188245; doi:10.1242/dev.204505)
Supplement: Supplementary information [file develop-152-204505-s1.pdf]

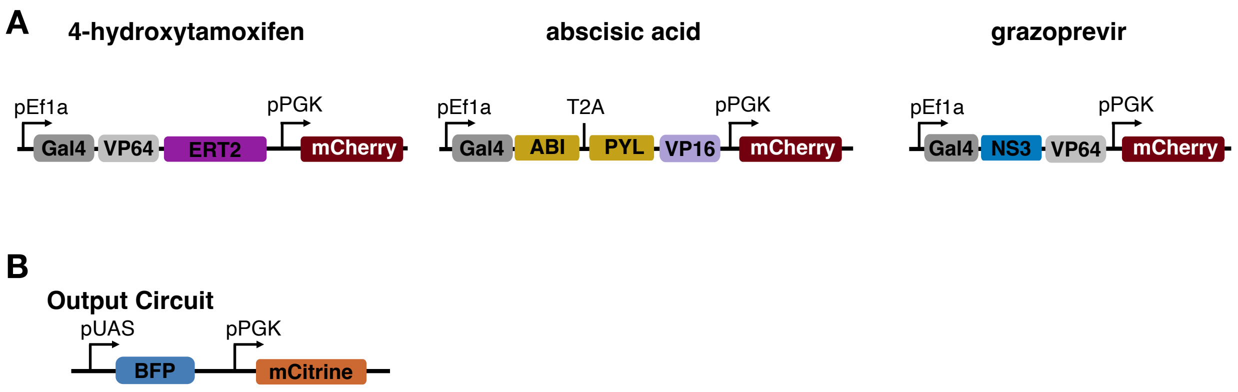

Fig. S1. Schematic Representation of Constructs. A) Schematic of each drug-inducible construct with a constitutively expressed mCherry. B) The output circuit, with TagBFP expression downstream of the (5X)UAS ybTATA promoter and a constitutively expressed mCitrine.

Development • Supplementary information

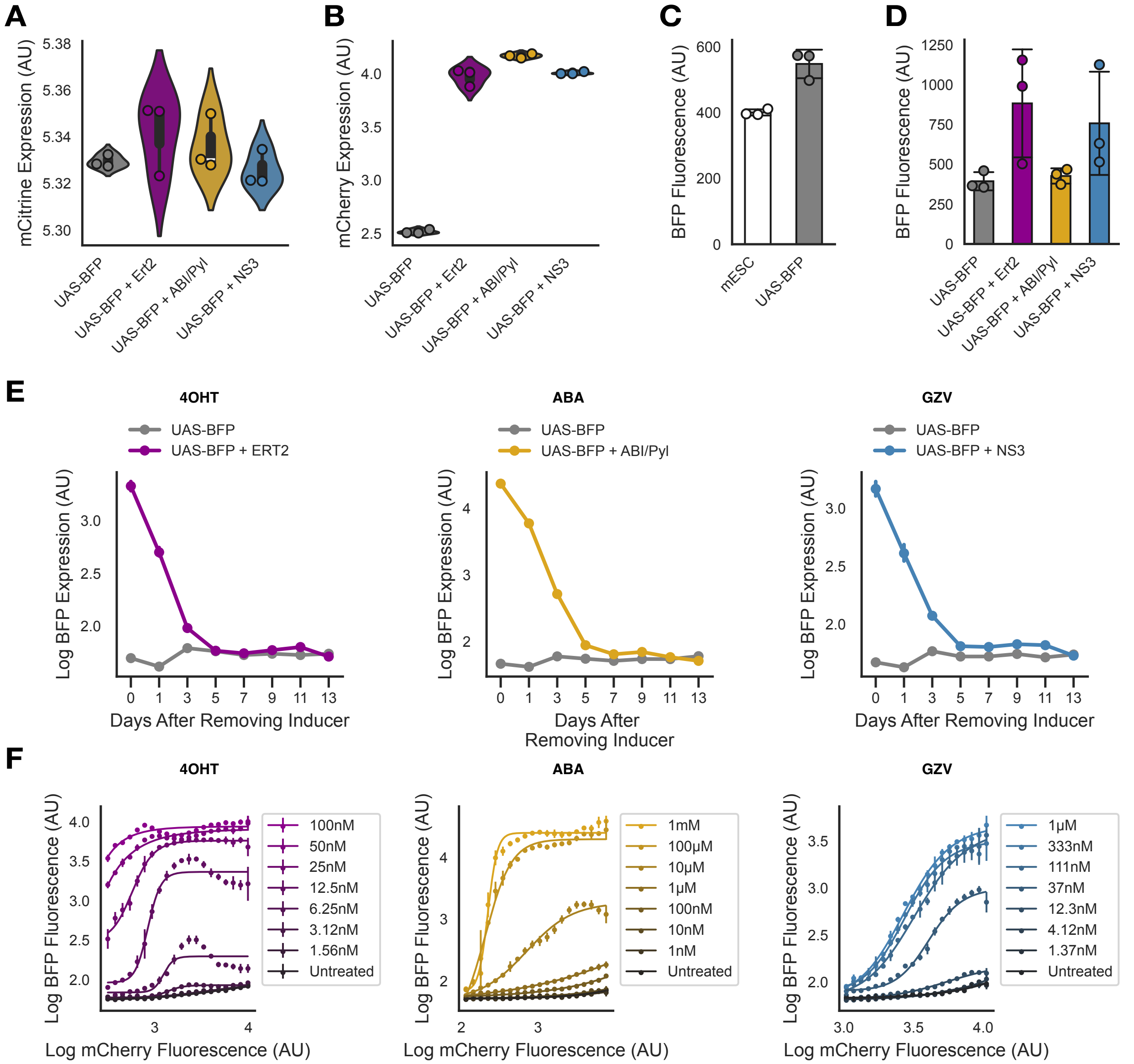

Fig. S2. Further characterization of small molecule-inducible systems in mESCs. A) mCitrine expression, representing copy number of the GAL4-UAS BFP output circuit, across the small molecule-inducible transcription factor expression lines. Data represented as violin plot to demonstrate absolute range of expression. B) mCherry expression, representing copy number of each transcription factor cassette, across the small molecule-inducible transcription factor expression lines. Data represented as violin plot to demonstrate absolute range of expression. C) Baseline BFP fluorescence for mESC and UAS-BFP cells, indicating the basal leak of the UAS promoter in mESCs D) Baseline BFP fluorescence for each cell line used in Figure 1 incubated for 3 days in vehicle, indicating the level of basal leak in each system. E) Log BFP fluorescence as a function of days since inducer withdrawal. 4OHT was initially dosed

Development • Supplementary information

at 100nM before washout, ABA at 1mM, and GZV at 1μM. F) Log BFP fluorescence as a function of transcription factor expression (log mCherry fluorescence) at each dose of inducer tested in Fig. 1. A polyclonal population of cells expressing each transcription factor was generated and sorted for all mCherry positive cells, resulting in an approximately 1.5 log range in transcription factor expression. Cells were grouped into 20 equally spaced bins of mCherry fluorescence and then their mean BFP fluorescence was calculated. The resulting data points were fitted to a 4 parameter logistic function to model the dependence of payload production on transcription factor expression.

Development • Supplementary information

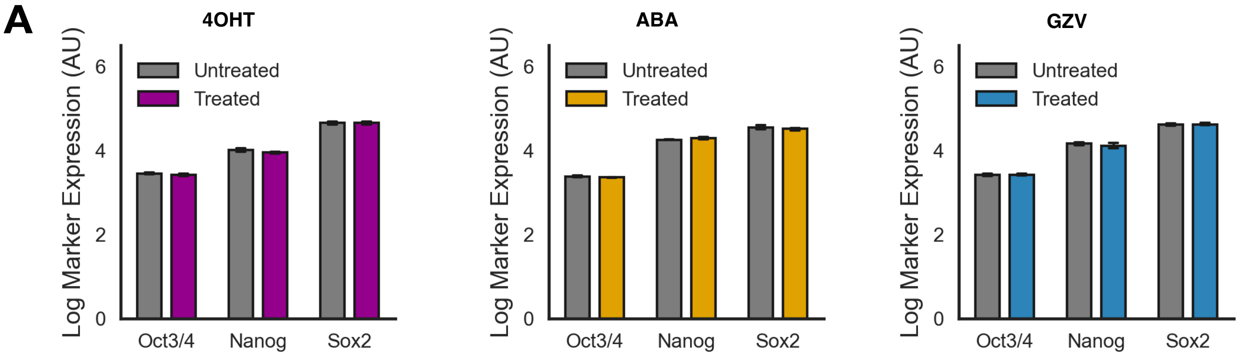

Fig. S3. The effect of small molecule inducers on the pluripotency state of mESCs. A) Wildtype mESCs were incubated with either 100nM 4OHT, 1mM ABA, or 1µM GZV for three days and then were fixed and stained for the indicated markers of pluripotency. Each graph displays the median level of fluorescent signal across 3 biological replicates for each of the 3 markers in cells treated with either vehicle or the indicated inducer.

Development • Supplementary information

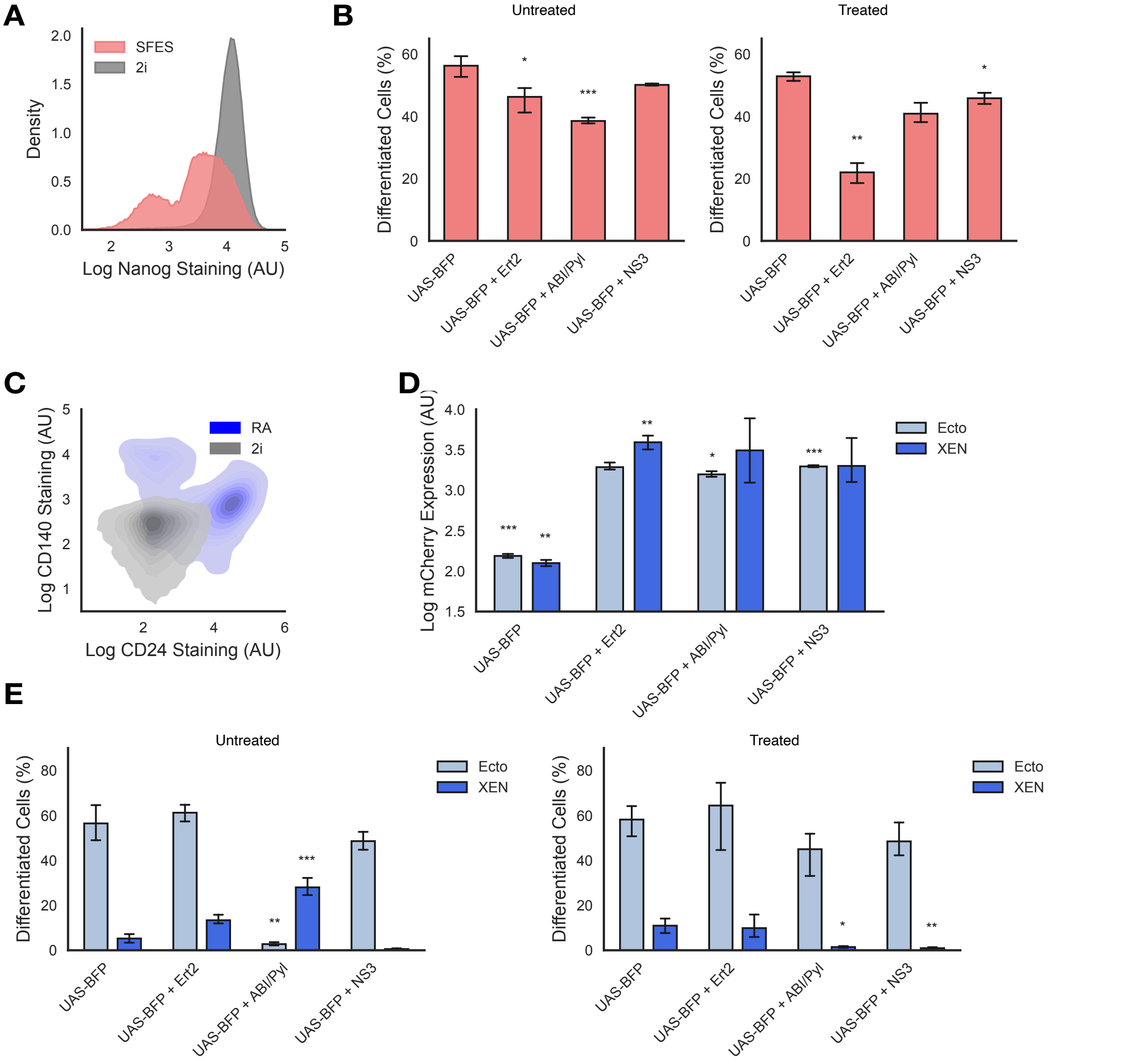

Fig. S4. Further characterization of small molecule-inducible systems across differentiation states. A) Kernel density estimate of Nanog-stained UAS-BFP cells cultured for 3 days in either 2i or SFES. B) Comparison of differentiation efficiency of EpiLCs (Nanog-) for each cell line used in Figure 2 after being treated with vehicle or inducer. Significance markers on the Untreated graph represent comparison to the same differentiation state in the UAS-BFP cell line, while significance markers on the Treated graph represent comparison to the indicated differentiation state of the indicated cell line in the Untreated condition. C) Two dimensional kernel density plot for cells cultured in either 2i or SFES + RA for 4 days and then stained for CD140 (PDGFRA) and CD24. D) Transcription factor expression (log mCherry fluorescence) in

Development • Supplementary information

each cell line used in the experiments in Fig. 2 as a function of cell identity after differentiation. All significance markers indicate comparison to ESCs (CD140-/CD24-) of the indicated cell line. E) Differentiation efficiency of each cell line treated with RA and either inducer vehicle (Untreated) or the max concentration of each inducer (100nM 4OHT, 1mM ABA, or 1μM GZV, Treated) for 96 hours. Significance markers defined as in B for Treated and Untreated graphs. Statistical indicators represent 'ns' = $p>0.05$, * = $p<0.05$, ** = $p<0.01$, *** = $p<0.001$.

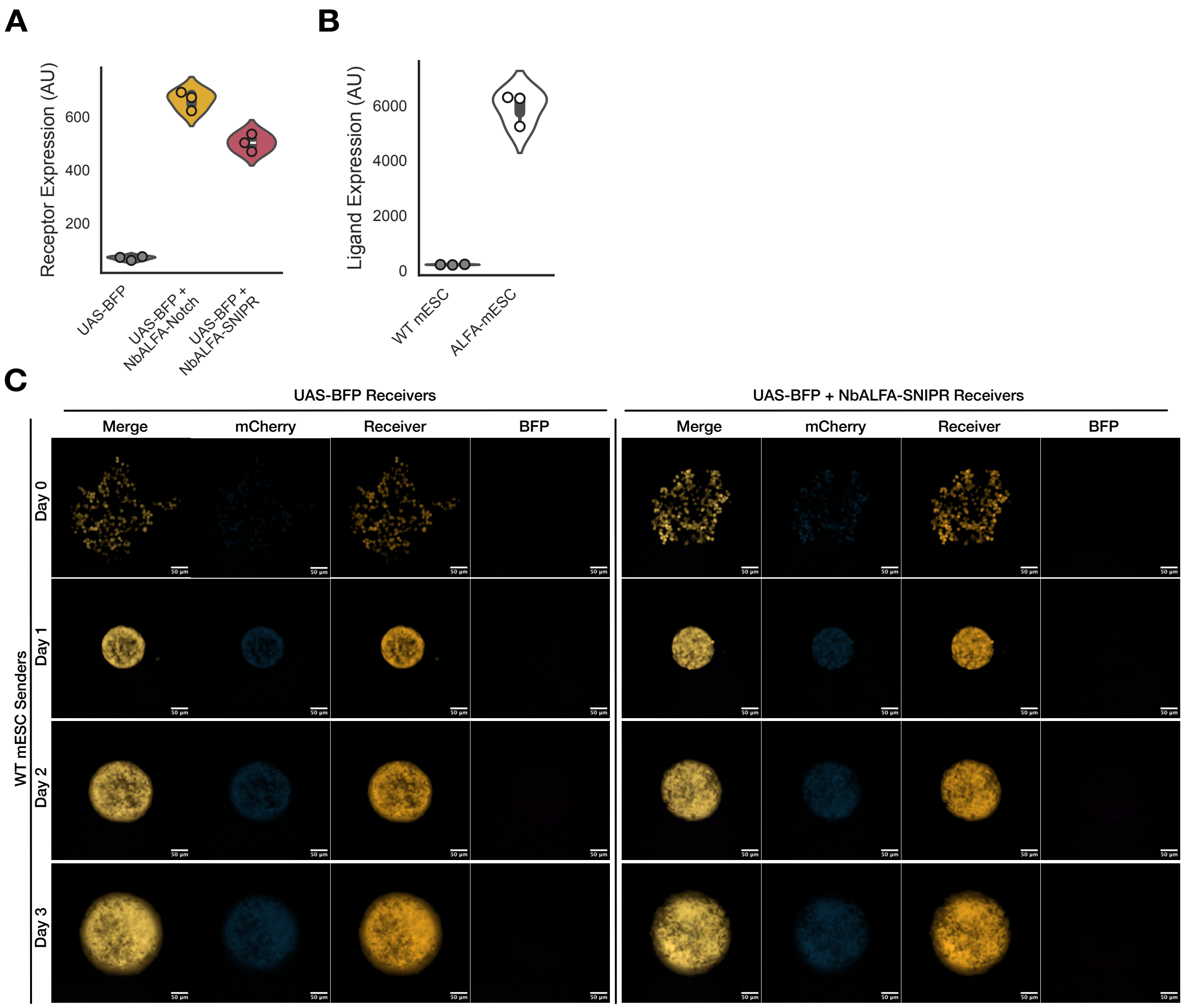

Fig. S5. Juxtacrine Receptor and Ligand Expression Levels. A) Comparison of median receptor expression levels across UAS-BFP, UAS-BFP + NbALFA-Notch, and UAS-BFP + NbALFA-SNIPR-expressing mESCs as assessed by surface expression using anti-Myc staining. Data represented as violin plot to demonstrate absolute range of expression B) Comparison of median ligand expression levels between wildtype mESCs and ALFA-mESCs as assessed by surface expression of ALFA-tag using anti-ALFA-tagstaining. Data represented as violin plot to demonstrate absolute range of expression. C) Co-cultured mESC sender cells with either UAS-BFP or UAS-BFP + NbALFA-SNIPR receiver cells in 2i at a 1:1 ratio in a low-attachment plate form spheres. Images taken every 24 hours with confocal microscopy. Single slices from the middle of the Z-stack are shown. Scale bars, 50μm.

Development • Supplementary information

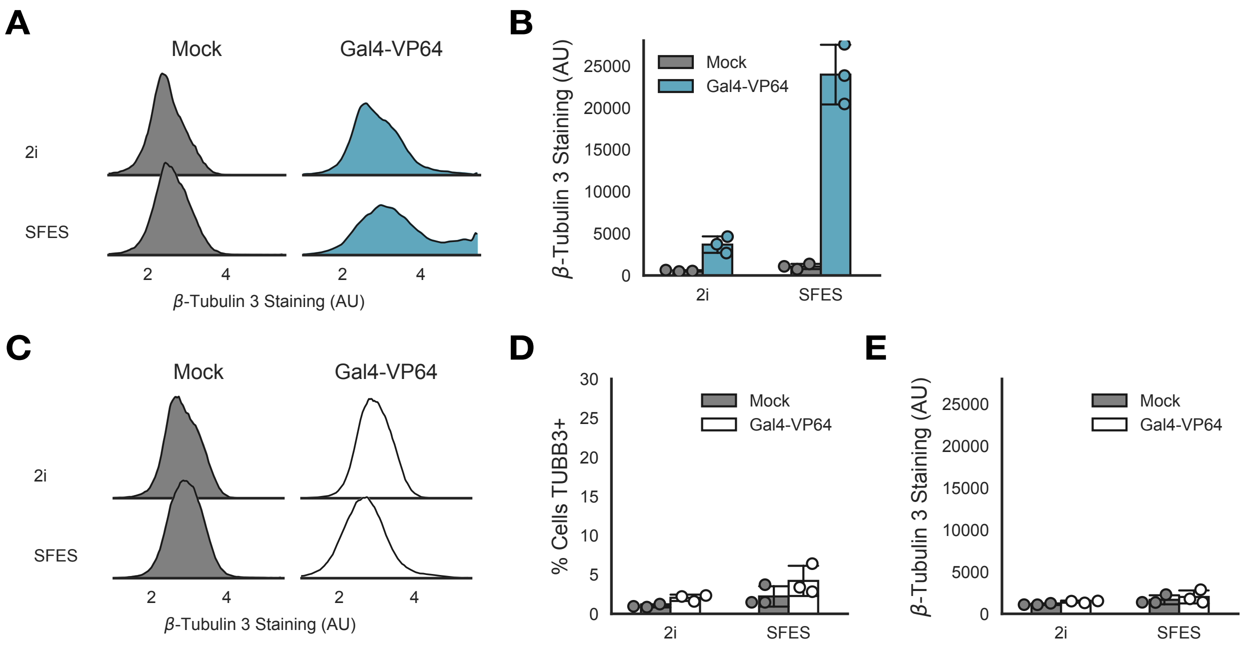

Fig. S6. Quantification of UAS-Ngn2. A) Population density plots comparing class III beta tubulin staining in UAS-Ngn2 in either mock transduced or Gal4-VP64 transduced conditions grown in either 2i or SFES media. B) Mean class III beta tubulin staining in UAS-Ngn2 mock transduced or transduced with Gal4-VP64 and cultured in either 2i or SFES. C) Population density plots comparing class III beta tubulin staining in wildtype mESCs in either mock transduced or Gal4-VP64 transduced conditions grown in either 2i or SFES media. D) The percent of wildtype mESCs that stained positively for class III beta tubulin (TUBB3) as assessed by gating on the 99th percentile of class III beta tubulin staining in mock transduced mESCs cultured in 2i. E) Mean class III beta tubulin staining in wildtype mESCs.

Development • Supplementary information

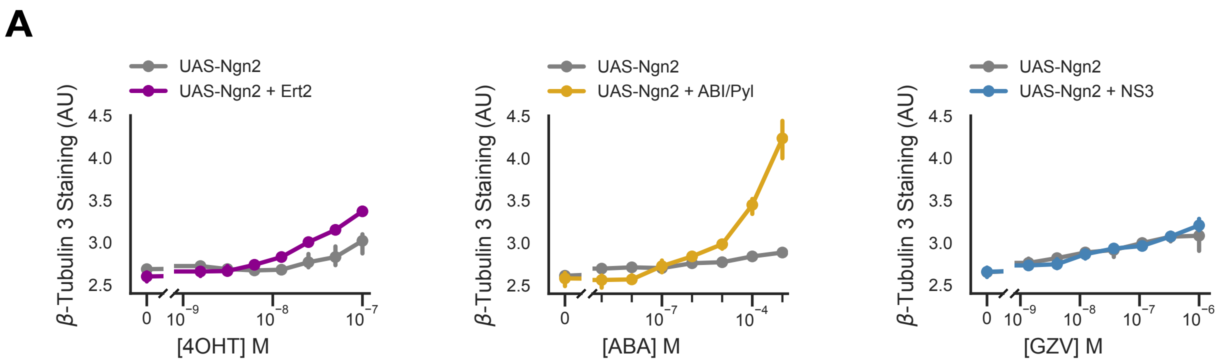

Fig. S7. Class III β-tubulin staining for small molecule driven UAS-Ngn2. A) Median class III β-tubulin staining for each inducible driven UAS-Ngn2 system as measured by flow cytometry.

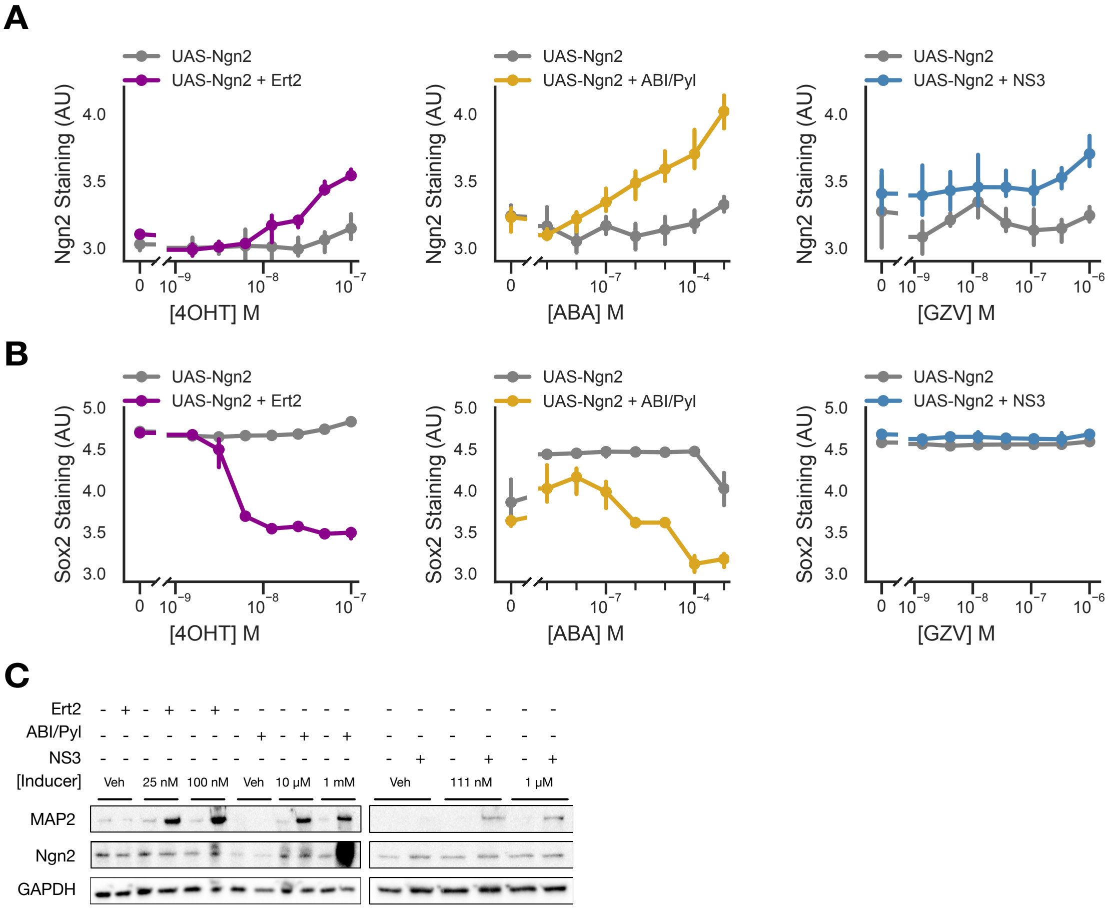

Fig. S8. Neuronal marker validation. A) Median Ngn2 staining for each inducible driven UAS-Ngn2 system as measured by flow cytometry. B) Median Sox2 staining for each inducible driven UAS-Ngn2 system as measured by flow cytometry. C) Immunoblot for MAP2, Ngn2, and GAPDH as a loading control for each inducible driven UAS-Ngn2 system. Comparisons are between each inducer at two doses or vehicle in UAS-Ngn2 cells or UAS-Ngn2 cells with the indicated inducible system. Each 6 lanes from left to right represent Ert2/4OHT, ABI/Pyl/ABA, and NS3/GZV respectively. Immunoblot is from a single experiment.

Development • Supplementary information

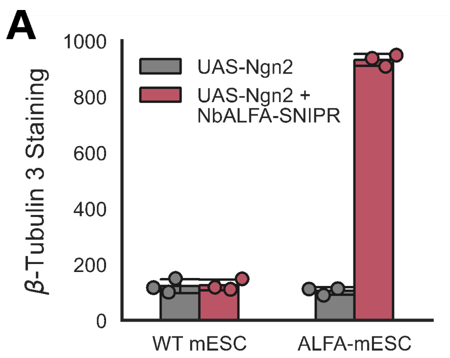

Fig. S9. Quantification of NbALFA-SNIPR UAS-Ngn2. A) Median class III β-tubulin staining in UAS-Ngn2 and UAS-Ngn2 + NbALFA-SNIPR after co-culture as measured by flow cytometry.

Development • Supplementary information

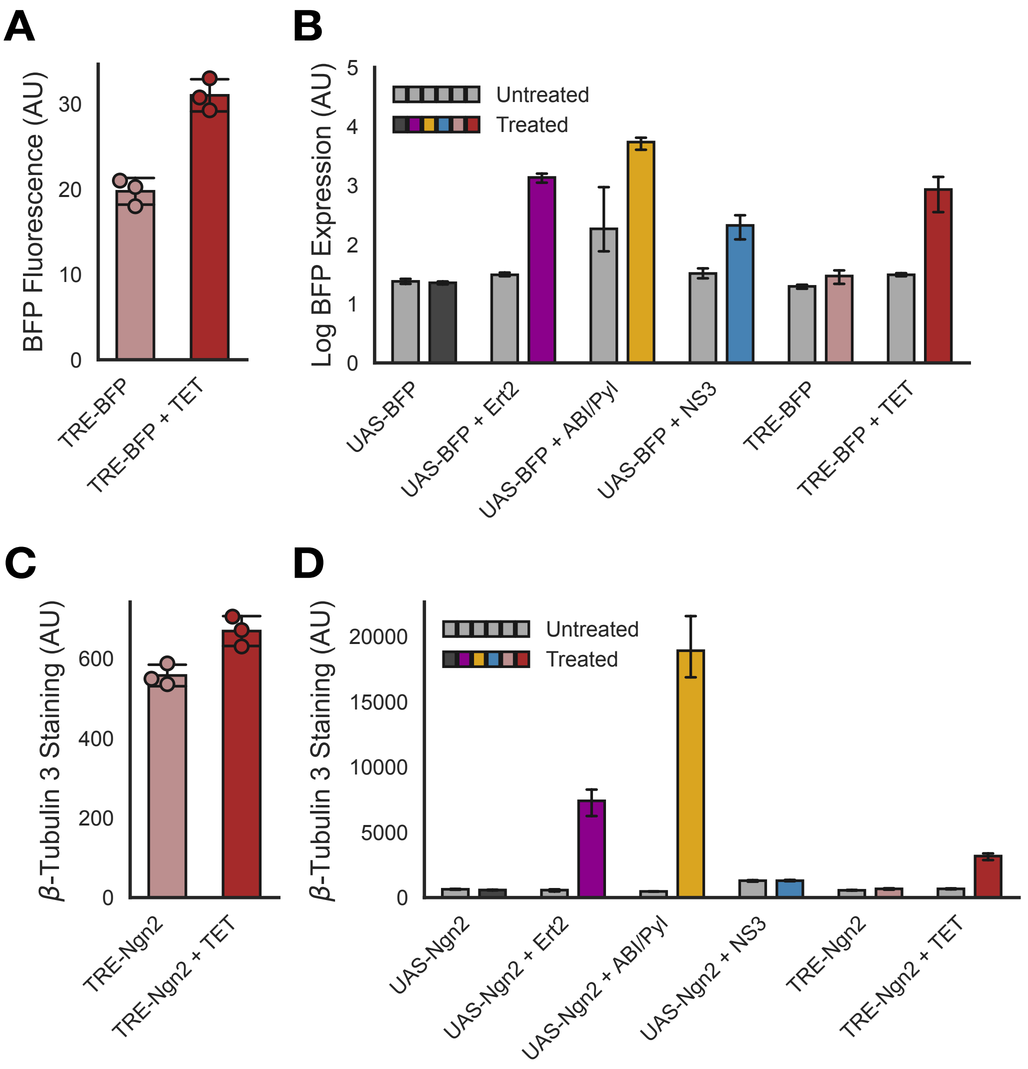

Fig. S10. Comparison of small molecule inducible systems to TetR in driving BFP expression and Ngn2 differentiation. A) Comparison of BFP expression when treated with vehicle (DMSO) to TRE-BFP + TET when treated with the inducer doxycycline (1μM) as measured by flow cytometry. B) Comparison of log BFP expression across inducible systems using 100nM 4OHT, 1mM ABA, or 1μM GZV and 1μM doxycycline. C) Median class III β-tubulin staining comparing TRE-Ngn2 when treated with vehicle (DMSO) to TRE-Ngn2 + TET when treated with the inducer doxycycline (1μM) as measured by flow cytometry. D) Comparison of median class III β-tubulin staining across inducible systems.

Table S1. DNA constructs

Available for download at

https://journals.biologists.com/dev/article-lookup/doi/10.1242/dev.204505#supplementary-data

Development • Supplementary information
