# Supplementary material for: Small molecule- and cell contact-inducible systems for controlling expression and differentiation in mouse embryonic stem cells
Source: Development. 2025 Jun 10;152(11):dev204505. doi: 10.1242/dev.204505 (PMC12188245; doi:10.1242/dev.204505)
Supplement: Supplementary file 1 [file develop-152-204505_review_history.pdf]

Small molecule- and cell contact-inducible systems for controlling expression and differentiation in mouse embryonic stem cells

Sarah S. Soliman, Devan H. Shah, Hana El-Samad and Zara Y. Weinberg
DOI: 10.1242/dev.204505

Editor: Matthias Lutolf

Review timeline
Original submission: 4 November 2024
Editorial decision: 20 May 2024
Resubmission: 7 November 2024
Accepted: 31 March 2025

Original submission

First decision letter

MS ID#: DEVELOP/2024/202881

MS TITLE: Small molecule and cell contact-inducible systems for controlling expression and differentiation in mouse embryonic stem cells

AUTHORS: Sarah S. Soliman; Devan H. Shah; Hana El-Samad; Zara Y. Weinberg
ARTICLE TYPE: Research Article

Dear Dr. Weinberg,

I have now received all the referees' reports on the above manuscript, and have reached a decision. The referees' comments are appended below, or you can access them online: please go to BenchPress and click on the 'Manuscripts with Decisions' queue in the Author Area.

As you will see, the referees express considerable interest in your work, but have some significant criticisms and recommend a substantial revision of your manuscript before we can consider publication. If you are able to revise the manuscript along the lines suggested, which may involve further experiments, I will be happy receive a revised version of the manuscript. Your revised paper will be re-reviewed by one or more of the original referees, and acceptance of your manuscript will depend on your addressing satisfactorily the reviewers' major concerns. Please also note that Development will normally permit only one round of major revision. If it would be helpful, you are welcome to contact us to discuss your revision in greater detail. Please send us a point-by-point response indicating your plans for addressing the referees' comments, and we will look over this and provide further guidance.

Reviewer 1 Advance Summary and Potential Significance to Field:

In this manuscript, Soliman et al. implement several synthetic biology tools in mouse embryonic stem cells (mESCs). Some had previously been shown to be functional in mESCs (ERT2/4-OHT, ABI/PYL/abscisic acid, and synNotch), whereas others are characterized in this cell type for the first time (NS3/grazoprevir, SNIPRs). All had been previously developed and characterized in other cell types.

The authors demonstrate how these tools can be used not only to overexpress fluorescent reporters, but also to drive neuronal differentiation of pluripotent cells in non-permissive

© 2025. Published by The Company of Biologists under the terms of the Creative Commons Attribution License (https://creativecommons.org/licenses/by/4.0/).

conditions by selectively inducing the pro-neural transcription factor Neurogenin2 (the neurogenic potential of which in pluripotent cultures was previously reported).

This article represents an important stepping stone in the expanding field of synthetic developmental biology, demonstrating functionality of several synthetic biology technologies in mESCs for the first time, and comparing different tools, thus encouraging researchers who make use of these cell lines to incorporate these tools into their research.

It is however unclear to me whether the cell lines described in this manuscript will be a useful resource for the community: the cell lines described are polyclonal and haven't been characterized in depth in differentiation assays, nor used to generate mice, so the transgenes utilized may be subject to silencing or positional effects. The authors did not characterize the effects of abscisic acid and grazoprevir on pluripotency and differentiation of wild-type cells, potentially limiting the usefulness of tools relying on these drugs. Finally, the synNotch system described in this article underperforms the "SyNPL" system established in mESCs and published in this journal in 2022 (DOI: 10.1242/dev.200226), where clonal lines were generated using a different synNotch receptor, ligand and transgene delivery method.

That said, if the authors were prepared to carry out further characterization of the systems, this would considerably strengthen this manuscript and help make these technologies widely attractive to the developmental biology community.

Reviewer 1 Comments for the Author:

General comments:

Part of the innovation introduced in this article is the ability to use lentiviral vectors followed by cell sorting of polyclonal lines expressing similar levels of fluorescent transgenes, to generate and screen cell lines in a faster manner than deriving clonal lines and comparing their behaviour. However, the ability of these polyclonal lines to sustain quasi-homogeneous transgene expression in transgene-independent differentiation (or in chimeric embryos) was not tested. Considering the strength of mESCs as a developmental model lies the modelling of differentiation events, and the generation of mouse lines, I believe it is important to either show that this method for deriving transgenic cells does produce silencing-resistant cell lines, or to make a strong statement that this method is useful for initial technology validation and iteration, but that clonal cell lines might be necessary for applications beyond pluripotency, (e.g. where lentiviral delivery vs landing pads are discussed on page 11).

Regardless of the path chosen to address the comment above, it would be important to show whether the 5 synthetic biology technologies established in mESCs work in a simple differentiation assay, (e.g. SFES [aka N2B27] neural differentiation, or in "LIF withdrawal", or an alternative assay), or whether they are functional only in 2iLIF/pluripotent cells. Any in vivo data (e.g. in chimeric embryos) would be a huge bonus but not essential in my view.

Most technologies described are tested by the authors measuring percentages of induced cells, but these percentages aren't calculated by using a negative control to set a gate for positive/negative cells in flow cytometry or image analysis, but rather by fitting a two-component Gaussian mixture model to the distributions.
The authors' previous work (doi: 10.1101/2021.04.20.440678) describes this as an appropriate model when dealing with bimodal distributions, but many of the distributions displayed in this manuscript do not appear bimodal (e.g. Fig.2, potentially Fig. S3 – the Gal4-VP64 SFES sample is saturated/truncated at its maximum so the shape of the distribution can't be judged), and for many figures distributions aren't shown at all.
The authors are undoubtedly better placed than me in judging whether their data is bimodal, but I would nonetheless like to see percentages calculated using a negative control for each percentage plot, at the very least in the supplemental information. I would also like to see distributions in supplemental for experiments where they are not shown.

Although 4-OHT has been widely used over the years in mESCs, abscisic acid and grazoprevir are less characterized. What is their effect on the expression of naïve pluripotency, primed

© 2025. Published by The Company of Biologists under the terms of the Creative Commons Attribution License (https://creativecommons.org/licenses/by/4.0/).

pluripotency and early differentiation genes? Do these drugs affect essential processes, such as differentiation or proliferation?

I found the combination of quantitative data and IF panels particularly useful in the programmed neuronal differentiation experiments. For example, the IF illustrates the fact that mESCs are weakly stained by the Tuj1 antibody (which has been seen by others too), but that the levels are very low compared to differentiated neurons.
However, in my view there is a mismatch between percentages of differentiated neurons displayed in quantitative data panels and what the images are showing, and I fear this may be due to the inclusion of these weakly stained cells in some calculations. For example, in Figure 4C, first panel almost 20% of mESCs are reported to be “differentiated”, but these are pluripotent cells with weak Tuj1 staining, not differentiated cells. I think gating for positive cells by using a positive control (e.g. mESCs differentiated in SFES/N2B27 for long enough – more on this in the Specific Comments section) would ensure that the quantification is more accurate.

I found the term iN quite confusing, as this is commonly used in the stem cell and reprogramming fields to refer to induced neurons directly programmed from differentiated cell types (e.g. fibroblasts). Whilst not technically wrong, as these neurons are induced too, I would consider a change of name. At the very least, I don’t think pluripotent cells carrying the UAS-Ngn2 transgene should be called iNs, as these are not induced neurons.

Specific comments:
Figure 1: How leaky are the systems? Please include a wild-type control in panel E (and ideally in panel C), and comment on the impact of leakiness (if present) for the broader applicability of the systems.

Figure 1D: what concentration of drugs were used for this panel? Please include the information in the legend.

Figure 1: Would it be possible to use the same y-axis in panel C for all three systems, and the same x-axis in panel E for all three systems, to make them easier to directly compare to one another? Or does this compress distributions too much?

Page 2: “The number of tools available to synthetically rebuild development are lacking in terms of the available breadth of small molecule and cell contact-inducible systems and the rapid iterability in which these systems can be incorporated into developmental models.”
This is a very strong statement, which could do with some contextualization. In Drosophila, for example, there are quite a few neighbor-inducible systems (reviewed in this journal a few months ago: DOI: 10.1242/dev.201955).

Page 2: it would be useful to make it clear that ERT2/4-OHT has been used for over a decade in the mESC community to drive nuclear translocation of transcription factors (e.g. Cre, Nanog, etc.). I think this would make the tool more appealing to the community as the technology is familiar.

Page 3: What “similar” levels of Citrine and mCherry were used in sorts? Very high? Medium? Low? Please include a distribution and gate used in the sort in supplemental.

Related to the previous comment: do the authors know if the systems only work when sorting for those levels of transgene expression? E.g. if they sorted on low expressors, is there no inducibility for high expressors as the baseline levels are too high? Or if they sorted on high expressors, is there a minimum level of transgene expression required for system functionality? This question applies to all cell lines generated this way, including synNotch and SNIPR.

Page 5: “We sought to improve juxtacrine inducible signaling in mESCs”
The authors do not later comment on whether improvement on the existing mESC system (SyNPL) was achieved. In my view it wasn’t, which may be due to a number of reasons (different signal/receptor, lack of clonality, delivery method). The comparison between the authors’ synNotch mESC, SNIPR mESCs and SyNPL is important for the DevBio community, and should be discussed in the Discussion section.

© 2025. Published by The Company of Biologists under the terms of the Creative Commons Attribution License (https://creativecommons.org/licenses/by/4.0/).

Page 5: “We hypothesizes that the functional differences between SNIPRs compared to the original synNotch might position SNIPRs as better suited for rapid iteration in engineering mESCs.”.
The text doesn’t make it clear to me why SNIPRs more suited than the original modular synNotch receptor, and I didn’t see evidence of iteration in this manuscript.

Page 5: “In addition to optimizing for rapid iteration”. It was unclear to me what this optimization was.

Page 5: the term “Sender Cells” is used, but the readership of this journal may be unfamiliar with it, I would recommend explaining it.

Page 5: Culture of cells as suspension spheres is described. Is it possible to see a picture of these, to understand size and 3D structure? Is there a lumen? If not, are there necrotic cells in the middle after 3 days of culture?

Related to the previous comment, Is it possible that synNotch and SNIPR would work better in adherent monolayers, where previously published SyNPL receiver mESCs showed >90% induction?

Figure 2C: what causes the broad distribution of tagBFP in activated SNIPR cells? Is there a correlation between receptor levels and tagBFP levels? Or between number of sender neighbors and tagBFP levels? Do all receivers express the receptor, and do all senders express the ligand? What are the practical implications of this broad distribution for users who might want to use SNIPRs to express functional transgenes?

Page 6: Do the authors have any data to back up the claim that the neurons are glutamatergic? If not, I would recommend not using this term, the results are just as impressive.

Page 6: what constitutive promoter was used for Ngn2? (info not found in methods)

Page 7: “Wild-type ESCs did not differentiate” (Fig S3C). This makes little sense to me. SFES medium (N2B27) is used to drive neural induction from mESCs, with most cells converting within 6 days of differentiation from 2iLIF, which is the length of this experiment (Mulas et al 2019 Development, doi: 10.1242/dev.173146).
Why do the authors think this is the case? Do the wild-type cells have any issues? Do they simply need more time?

Figure 3D: Are these cells cultured in 2iLIF or in SFES (N2B27)? If this image is not representative of the dish (which I presume is the case due to the disparity between % differentiated in the graph and all cells being Tuj1+ in the image) the authors should clarify this in the legend.

Figure 4 and Figure S4: all three systems seem to work if the IF is representative, see general comment above re: quantification. With regards to the increased differentiation seen with 4-OHT and GZV, the intensity levels in Fig.S4 are still very low compared to the levels I would expect to see in actual neurons (at least 2 log difference vs uninduced), so they may not be particularly relevant. Distributions of signal in single cells would go a long way in clarifying this.

Figure 5A: Add mCherry transgene to sender cell diagram for clarify.

Page 11: “Each of the components demonstrated in our study can readily be used on their own or in combination in applications to further our understanding of developmental principles.”
The authors have not demonstrated this is the case, and should explain this is a theoretical statement (or that it has been done in other systems if this is the case).

Page 11: “For example, what is the minimal and maximal amount of a reprogramming transcription factor needed to be in the “ON” state for differentiation to occur?”
Confusing, as “reprogramming transcription factors” (Oct4, Sox2, Klf4, c-Myc) maintain pluripotency (broadly speaking at least). “Pro-differentiation transcription factor” may be a more suitable term.

© 2025. Published by The Company of Biologists under the terms of the Creative Commons Attribution License (https://creativecommons.org/licenses/by/4.0/).

Page 11: “The different mechanisms of the inducible systems used here might suggest an answer, whereby the only system that requires de novo synthesis of its transcription factor was also the only system unable to stimulate differentiation.”
I find this sentence very hard to understand. If there is an answer suggested by this paper, it should be discussed. What is “the only system that requires de novo synthesis of its transcription factor”? Is this referring to SFES (N2B27) differentiation of wild-type cells? If so, I think this is an issue that needs to be addressed, see comment above.

Page 11: “Future work should explore differences in overall payload production between these inducible systems.”
Isn’t this what Figure 1C shows?

Page 11: “The tools we present in this manuscript are essential for enabling the next generation of synthetic developmental biology”
I think this is an overstatement.

Methods:
-What mESC line or lines were used? Are they male or female? Were they karyotyped? Tested for mycoplasma contamination?
-“Ngn2-dependent direct differentiation” section: “Cells were incubated for at least six days with media refreshed every two days.”
The length of incubation in SFES (N2B27) plays a major role in the percentage of Tuj1+ cells in the dish. Can the authors be more specific with regards to the length of incubation in SFES?
-Are confocal data displayed as maximum intensity projections or single z-slices?

Minor:
Fig.1C, E axes are labelled log, Fig 1D axis is labelled log10, which are the same thing. Ideally fix for consistency.

Page 1: I don’t think reference 9 is suited to the sentence it is used in, other articles, including reviews cited in the next sentence, would be more appropriate here. Reference 9 is an important paper, relevant to this study, and should certainly be cited elsewhere in this article.

Page 5: “previous implementations of this system in mESCs required single site genomic integration and subsequent single cell sorting and clone selection to achieve functional engineered cells”.
The functional cells used in this study were not subject to single cell sorting.

Reviewer 2 Advance Summary and Potential Significance to Field:

This is good work, that builds and demonstrates tools for the control of gene expression in mESCs. BUT I am not sure that Development is the right journal for this paper. Development exists mostly to publish advances in understanding of the development and regeneration of multicellular organisms. This paper does not add anything to our understanding of development; rather it presents information on the use of specific tools to control expression mainly of reporter genes in mESCs. The only 'developmental' thing was a proof of concept in control of differentiation, which did not add to our knowledge about this differentiation but merely showed that a tool behaved as expected. I would have thought the paper a much better fit to, for example, ACS Synthetic Biology (which is where this reviewer's own lab publishes this kind of paper).

Reviewer 2 Comments for the Author:

Overall:
This is good work, but I am not sure that Development is the right journal for this paper. Development exists mostly to publish advances in understanding of the development and regeneration of multicellular organisms. This paper does not add anything to our understanding of development; rather it presents information on the use of specific tools to control expression mainly of reporter genes in mESCs. The only 'developmental' thing was a proof of concept in control of differentiation, which did not add to our knowledge about this differentiation but merely showed

© 2025. Published by The Company of Biologists under the terms of the Creative Commons Attribution License (https://creativecommons.org/licenses/by/4.0/).

that a tool behaved as expected. I would have thought the paper a much better fit to, for example, ACS Synthetic Biology (which is where this reviewer's own lab publishes this kind of paper).

Title:
The title is too general. You worked on one specific type of stem cell, so that specific type ('mouse embryonic stem cells') should be in the title. A much larger body of work would need to be justified for 'stem cells' without further qualification.

Abstract:
I recommend you avoid the word 'chassis' as it is synbio jargon that may not be understood, or may not be welcome, in the developmental biology community. 'Platform' would probably work as an alternative.

Introduction:
This was clear and referenced existing work well.

Results:
I appreciated the cartoons in Fig 1a,b and Fig 2a,b etc.. Fig 1c would be better if the y axes of all 3 graphs were the same, since it invites comparison between the 3 systems (they are very close to the same, but not quite). In Fig 1e, I think 'concentration' would be a more accurate term than 'dose' (there is no pharmacokinetics involved as this is a simple in vitro system). In Figs 3-5, the text labelling the scale bars is tiny – please repeat it in the figure legend.

Discussion:
Again, please avoid 'chassis' if you are accepted for Development (use of the term would be more acceptable in a synthetic biology journal, though I hate it even there for its implication of inertness). The discussion of the problems in the system needing de novo synthesis of its transcription factor was interesting (Discussion paragraph 3). The beginning of the final paragraph of the Discussion is clearly indefensible – your tools will no doubt be useful, but to claim they are "essential for enabling the next generation of synthetic developmental biology" is going way too far; any tools that operate in roughly this way could do the job. This is the language of grant/ promotion applications, not of scholarly papers. Your later comment "The tools presented here add to a growing toolkit..." is exactly right.

Final comment:
Again, I think this is good and useful work. I just think it would be better, and end up more used and cited, in a synbio journal.

Reviewer 3 Advance Summary and Potential Significance to Field:

In this paper, Soliman et al have tested a previously developed system for controllable induction of transcription factor in stem cells. The main impetus of their work is to develop an orthogonal system for small-molecule-mediated control of protein expression. They employed mESC as a testbed and transcription factor Ngn2 to provide proof of principle and showcase the potential of these tools in stem cell engineering.

Reviewer 3 Comments for the Author:

Comments:
1. In a recent paper from Khalil group published in science, a highly similar set of tools have been developed to enable small-molecule-mediated control of gene expression. The main idea in this paper was to develop a method to replace GAL4-DNA binding protein (and other non-human-derived elements) with zinc finger proteins to avoid immunogenicity and develop a more customizable DNA binding module. The authors have just used the same effector modules used in that paper with GAL4 as the DNA binding domain, which is at odds with previous effort to improve the GAL4-based tools. It is expected that the authors justify the rationale behind using GAL4 (the science paper shows GAL4 is immunogenic and not suitable for applications intended to be used in a clinical setting and also confirms a superior performance of zinc fingers over GAL4.).

© 2025. Published by The Company of Biologists under the terms of the Creative Commons Attribution License (https://creativecommons.org/licenses/by/4.0/).

2. The paper's introduction and discussion about developmental synthetic biology seems to be beyond the data provided. The authors just tested a modified GAL4-mediated system for the control of gene expression. It is expected that the authors remove these sections or clearly justify how this modified gal4 system for controlling gene expression can advance the field of developmental synthetic biology aimed at reconstructing developmental systems.
3. The authors used a log scale to represent the expression of fluorescent proteins. This can be misleading, making background activity less stressed out as it is.
4. Fig 3: In the differentiation studies, the authors used the transcription factor ngn2 as a proof of principle. They would need to show that this transcription factor could be titrated using protein analysis .
5. Fig 3: they authors are expected to stain against several hallmark features of neurons to confirm the identity of the resulting cells .
6. Fig3: The authors should also provide data showing that the resulting neurons have electrical and other intended physiological activities.
7. It's often the case, a transcription factor must be turned off after it has been triggered. Can the others provide data regarding the on-to-off dynamics of this system. If it can be turned off and how fast.
8. Fig 1 and 3: the authors must definitely compare the proposed toolset with tet system (or/and zinc finger system) and include the data in these figures. Compare: dynamic range, background activity, how fast the systems can be turned on and turned off.
9. Fig 4: The authors claim is to develop 3 different orthogonal systems for controlling gene activity in stem cells. However, 2 out of 3 approaches performed very poorly. How this can help as a tool for future applications.
10. Fig 4: mESC is not the right control group for this experiment. The authors should use mESC engineered with the circuits' responder layer. Then, they should add the same small molecules to the control groups with the responder layer only.
11. Fig 5: in this experiment mESC is not the right control group. mESC engineered with the responder layer and not having the synthetic receptor must be used as a control.
12. The data presented in this paper mostly lack statistical analysis. The authors are expected to analyze the data using the right statistical method.
13. It will be important to provide data regarding differentiation efficiency among engineered cells and discuss silencing if it occurs.

First revision

<u>Author's response to the reviewer's comments</u>

Please find attached our revised manuscript. We deeply appreciate the reviewers' helpful feedback. In addressing that feedback, we believe we have improved our study with reviewer-suggested experiments that further demonstrate the impact and intricacies of cell engineering in model developmental systems.

In summary, we have improved the manuscript by:

- Adding a new main text figure detailing the functioning of the inducible systems we chose across multiple model differentiations, further demonstrating their potential for impact in mESC studies of developmental biology.
- Adding multiple new supplementary figures that detail additional aspects of our inducible systems, including the basal activity of each system, their off-rate kinetics, their expression-level dependent function, the effect of each inducer on the pluripotency state of wildtype mESCs, and a comparison to the well established Tet-ON induction system.
- Adding representative images of some of our juxtacrine signaling systems producing BFP to better demonstrate the health of these cultures and better qualitatively describe the spatial nature of activation within them.
- Reworked the names of our cell lines to help readers more easily understand the control conditions used in each experiment and avoid overloading already existing terms like

© 2025. Published by The Company of Biologists under the terms of the Creative Commons Attribution License (https://creativecommons.org/licenses/by/4.0/).

"iNs".
- Revised how we calculate "on" and "differentiated" cells to be based on a percentile of signal in our control conditions instead of the Gaussian mixture model approach that reviewers rightly pointed out was misrepresenting our primary data, especially in the differentiation assays.
- Further characterized our small molecule-induced neurons through staining for Ngn2, MAP2, and Sox2. In particular, this characterization better highlighted the quantitative requirements of Ngn2 expression for inducing neuronal differentiation.
- Adjusted phrasing throughout to better highlight the value of our systems, better situate them in the existing toolbox of cell engineering in mESCs, and more appropriately state their potential impact.
- Point-by-point responses to further reviewer comments are included below.

We believe these additions and modifications significantly improve the manuscript, provide potential users of our systems a better understanding of their features and limitations, and better demonstrate how these systems function in studies of developmental biology by demonstrating their functionality across multiple cell types differentiated from mESCs. We hope the reviewers agree.

Thank you again for your consideration. We look forward to hearing your and the reviewers' thoughts on the revised manuscript. Cheers, Zara, on behalf of all authors

Point by point response where relevant:

1. Reviewer 1 raised concerns about the general usefulness of the cell lines generated from this study due to their polyclonality and lack of in depth characterization. We agree with the reviewer and see the primary toolset generated in this study as the plasmids and methods, not the specific cell lines. We have aimed to emphasize this throughout the paper by highlighting the "systems" themselves. We hope the Reviewer agrees that these plasmids are useful tools in their own right and as the basis for further development from other labs.

2. Reviewers 1 and 3 raised concerns about the systems, especially the molecules used for system induction, affecting pluripotency and their functionality during differentiation. We especially appreciated this concern and in responding, added an additional main text figure (now Figure 2) that we think dramatically strengthens the manuscript and helps to better serve the target audience in Development. In summary, we see no effect of any drug on basal pluripotency and we see successful system functionality across 3 differentiated cell types using differentiations our lab has been using in other ongoing projects. Importantly, we see differences in system functionality in each cell type, emphasizing the value of having multiple inducible systems to choose from when engineering mESCs that will be used in differentiation assays.

3. Reviewer 1 notes that our juxtacrine systems underperform the excellently described SynPL system previously published in Development. We agree and have revised our discussion of SynPL in the intro and Results and added a paragraph in the Discussion to highlight this existing toolkit. In short, we think showing the comparison of SynNotch to SNIPR in our testbed is the primary contribution of this juxtacrine portion of this work, and we hope that these findings can inspire future SynPL-like tools.

4. Reviewer 1 raised concerns about our Gaussian mixture model-based % activation/differentiated quantification when comparing to the primary data. We agree with the reviewer, and have revised our activation quantification throughout the manuscript. Now we use a threshold of the 99th percentile of signal in the negative control condition for each experiment as the definition of "off" or "undifferentiated". This also addresses this Reviewer's concern about misrepresenting the level of differentiation in our SFES only mESC condition.

5. Multiple reviewers noted concerns about nomenclature throughout the manuscript.

© 2025. Published by The Company of Biologists under the terms of the Creative Commons Attribution License (https://creativecommons.org/licenses/by/4.0/).

Reviewer 1 was concerned about our use of “iN”, while Reviewer 3 suggested that some of our “mESC” controls were not the proper controls to be used because we overloaded this term. We have relabeled throughout the manuscript so that ‘mESC’ only ever refers to untransduced lines. Lines expressing our BFP output circuit are now described as “UAS-BFP”, and lines formerly described as ‘iN’ are now referred to as ‘UAS-Ngn2’. We hope this resolves concerns about overloaded terms and further helps to indicate that proper controls were used throughout.

6. Reviewer 1 asked “How leaky are the systems?”. We have added Supplemental Figure 2C to show that each system has minimal leak.

7. Reviewer 1 asked us to highlight which drug doses were used across different figure panels. We have added this information throughout the text and in figure captions.

8. Reviewers 1 and 2 asked us to match the scale on Figure 1C and D. These data were recorded across two different flow cytometers, one of which was decommissioned during the course of these experiments, hence the different baselines shown in this figure initially. In order to make these data easier to interpret, we have normalized them to fluorescence of the UAS-BFP untreated control in each experiment and adjusted the figure caption to match.

9. Reviewer 1 suggested we highlight the decades-long use of tamoxifen-inducible systems in developmental biology. We appreciate this advice and have added the following to the text:

The first of these drugs is 4-hydroxytamoxifen (4OHT), that selectively modulates the nuclear availability of molecules fused to a sensitized variant of the human estrogen receptor Ert2. This 4OHT/Ert2 system has been widely used previously in mESCs and developmental biology in general, especially coupled with the Cre recombinase

10. Reviewer 1 asked for clarification regarding expression levels of our constructs. We have added Supplemental Figure 2A and 2B to show these expression levels for the small-molecule inducible systems and modified the existing expression level graphs in (now) Supplemental Figure 5. Reviewer 1 also expressed concern about the function of these systems across TF expression levels. To address this concern, we subjected an unsorted population of lentivirally-infected cells to our inducer treatments for each small molecule system and included these data as Supplemental Figure 2E. Unsurprisingly, transgene expression is directly correlated with transcription factor expression levels.

We have commented on this feature of the systems in the text. We did not do the commensurate experiment for SynNotch and SNIPR, but based on previous literature with these systems and our own previous experience with them, high expression of these juxtacrine-inducible systems is also conducive to higher payload expression.

11. Reviewer 1 noted the value of a comparison of our juxtacrine-inducible systems to the existing SynPL system. We have included this in our discussion and rephrased our claims about why we tested these inducible systems in the first place.

12. Reviewer 1 expressed concern about how we introduced SNIPRs. We have rephrased this introduction to better capture our thought process here:

Given our experience using SNIPRs in other heterologous systems, we suspected that SNIPRs would express better and have higher payload output and expression in mESCs as we have seen in other cell types.

13. Reviewer 1 correctly pointed out that we did not detail any optimization for rapid iteration. We have removed these claims from the text. We have opted to highlight where possible that the lentiviral transduction method we used to construct these lines is useful for rapidly prototyping circuits.

© 2025. Published by The Company of Biologists under the terms of the Creative Commons Attribution License (https://creativecommons.org/licenses/by/4.0/).

14. Reviewer 1 advised that we might want to better explain our Sender and Receiver cells to readers. We have added text to address this.

15. Reviewer 1 expressed concern about the spheroids we used in these studies. We have included images of these spheroids in Figure 3 to demonstrate the composition, spatial distribution, and lack of lumen in these structures. Reviewer 1 further asked if monolayers might lead to better activation compared to our spheroid cultures. From first principles, we propose 3D culture will result in higher surface area contact between adjacent cells, which we think is the primary driver of activation. We see very high activation (>80% of receiver cells) in our SNIPR spheroids, which we think supports this idea. We did not conduct a direct comparison of activation between spheroid and monolayer, but we should note that our juxtacrine Ngn2 induction experiments were conducted in monolayers. We aim to make no claim about efficacy of either culture method and simply leave each as an option to future users of this system.

16. Reviewer 1 commented on the broad distribution (>2 log) of BFP expression in our juxtacrine inducible cells. We agree with the reviewer that this is indeed strange, but that it matches with our previous experience using SynNotch and SNIPR in Jurkat immortalized cells and primary T cells. We suspect that this is an effect of the randomness intrinsic in cell-cell interactions, although we acknowledge that this is not present in all SynNotch-derived systems (eg, SynPL sees a broad activation range of ~2 logs, but 90% of cells are within 1 log of each other (Fig 3E-H). Given the quantitative requirements we point out for Ngn2-induced differentiation highlighted later in the paper, we have drawn attention to this characteristic of the juxtacrine inducible systems.

17. We have removed use of the modifier “glutamatergic” for our induced neurons since Reviewer 1 is correct and we have no evidence for that claim.

18. We have added detail indicating that we used a PGK promoter to drive Ngn2 expression. We should also note that all plasmids used in the study are included in our supplemental data deposition at Zenodo.

19. Reviewer 1 asks why our WT mESCs in SFES did not differentiate into neurons, citing Mulas 2019 as an example where this differentiation can be seen. We agree with the reviewer that the difference here is concerning. After carefully reviewing the protocol used in Mulas 2019, we suspect that culture differences (Mulas and colleagues cultured these differentiations on laminin, we cultured our cells on gelatin across all experiments), timing of SFES addition (Mulas et al seed cells directly into SFES, we seed into 2i and change into SFES one day later), and cell density differences (Mulas et al used very high cell density for their differentiations while we plated cells at much lower density). We hope these differences are enough to explain the difference in our results. We assessed our mESCs for pluripotency (Supplemental Figure 3) and they were highly uniform, our cultures appear highly uniform without the characteristic fibroblast-like spread in cultures that are differentiating at baseline, and we are successfully able to generate epiblast-like cells and ecto- and XEN-like cells from these cultures as indicated in Figure 2, so we don’t think there’s anything wrong with the cells. Mulas et al suggest that failure to differentiate could be due to low-quality SFES. Although we think this is unlikely given the markers of cell health and viability noted above, we accept that this may be a possibility as we developed our mESC culture practices almost exclusively from Mulas 2019.

20. We have noted the media used in different differentiation conditions and clarified that images in Figure 5 are NOT representative, hence our use of the flow assay for quantitation.

21. Reviewers 1 and 2 had multiple issues with our discussion which we have thoroughly reworked, removing or rephrasing the lines they took issue with.

22. We have added details in the methods section that Reviewer 1 inquired about including

© 2025. Published by The Company of Biologists under the terms of the Creative Commons Attribution License (https://creativecommons.org/licenses/by/4.0/).

the mESC line used, specifying the duration of culture for neuronal differentiation, and describing how our confocal images are depicted.

23. We have made sure all "log" axis labels are labeled consistently.

24. We have redistributed citations in the introduction per Reviewer 1's suggestion.

25. We have corrected our description of SynPL to highlight that receiver genetic constructs were integrated randomly instead of site-specifically.

26. Reviewer 2 expressed concern about the fit of our study for Development. We agree with the reviewer's assertion that ACS Syn Bio or a similar journal would also be a good fit for this work, but we think that Development is an ideal target for this work as we hope that these systems will prove useful to a wide variety of developmental biologists who read Development but not perhaps other synthetic biology journals. Further, Development has a history of publishing useful synthetic biology tools (for example, SynPL which has been referenced throughout the text and in these reviews, as well as other papers like Vigano et al 2021, Aughey et al 2019, Wang… Eiraku 2024, Ell…Pyrowolakis 2024, etc). As part of reworking the text, we have tried to emphasize this perspective and remove as much synthetic biology "jargon" as possible. In the end, we leave the decision of journal fit to the editor.

27. We have added "mouse embryonic" to the title to better represent the work we have done, as suggested by Reviewer 2.

28. We have added scale bar descriptors to legends as requested by Reviewer 2.

29. We have prioritized using "concentration" instead of "dose" where possible.

30. Reviewer 3 asked that we justify our use of GAL4 instead of something like the synZFTR UAS that featured in Li, Israni 2022, specifically saying "*It is expected that the authors justify the rationale behind using GAL4.*" We have addressed this in the text as follows:

The GAL4 UAS-ybTATA system has not been used in mESCs and therefore it would represent an expansion to the orthogonal transcriptional regulatory elements toolkit. Although there are a multitude of orthogonal transcriptional elements (including the well described synZiFTR system), we selected the GAL4 UAS because of its broad compatibility with existing synthetic biology tools, including our lab's own Mammalian Cloning Toolkit (MTK)

We further appreciate Reviewer 3's concerns about immunogenicity, but we think this is likely to present minimal issue with these systems. In immunogenicity scoring prediction used in Li, Israni 2022, the GAL4 DNA binding domain scores only modestly worse than any of the zinc finger domains tested (4% of 9mers above the arbitrary cutoff of 0.23576 compared to 0% for all the zinc finger domains), and scores similarly to the native protein Egr1 (2% of 9mers scoring above the arbitrary cutoff). Further, from preclinical reports of Clinical Trial NCT06186401, which uses a GAL4 DNA binding domain as part of its engineered cell payload, our collaborators report as yet no signs of immune reaction to these engineered cells. In sum, we think the likelihood of immunogenicity of this system is low, less relevant for cultured cell studies which is a major use of mESC lines, and that the potential immunogenicity of the GAL4-DBD is outweighed by the value of adding an additional orthogonal transcriptional regulation system.

31. Reviewer 3 notes that "*The data presented in this paper mostly lack statistical analysis. The authors are expected to analyze the data using the right statistical method.*". We have added statistics in several figures, particularly in Figure 2 and its corresponding Supplemental Figure 3. However, we have opted not to include statistical comparisons in many of the figure panels throughout the paper. We believe that we have a philosophical difference of opinion from Reviewer 3 with regards to the presentation of statistics and their value throughout the scientific literature. We agree with Goedhar t 2018 that p values are

© 2025. Published by The Company of Biologists under the terms of the Creative Commons Attribution License (https://creativecommons.org/licenses/by/4.0/).

overreported throughout the scientific literature leading to both misinterpretation of their significance and also leading to a reduction in their value. Throughout our manuscript, many comparisons have large effect sizes (for example, Figure 1D, 2D, 3E, 5C, and 6C) with means distantly far apart and error bars that clearly do not overlap - we think including statistical comparisons and reporting p values in these cases does a disservice to the reader and to the literature in general.

32. Reviewer 3 expressed concern that our referencing synthetic development was beyond the scope of this paper. In our Discussion rewrite, we have sought to highlight how the addition of another orthogonal system to mESC engineering workflows is applicable across both developmental and synthetic developmental biology and we hope this satisfies the reviewer's concern.

33. Reviewer 3 is concerned that the log scale we use obscures baseline leak. We have added Supplemental Figure 2C to describe baseline leak in our system. In general, the log scale is a better representation for both the concentration-response graphs we show as well as for visualizing distinct populations in the histograms we show. We hope demonstrating minimal leak is sufficient to address the reviewer's concern.

34. Reviewer 3 expressed several concerns about our induced neurons including the titratability of Ngn2 expression, staining for more neuronal markers, and demonstrating the electrical properties of the neurons. We thank the reviewer for this suggestion as staining for Ngn2 (Supplemental Figure 9) provided very useful insight into the quantitative requirements of Ngn2 expression for differentiation. We added staining for MAP2 (a positive marker of neuronal identity) and Sox2 (a negative marker of neuronal identity) to further convince readers of the identity of these cells. Unfortunately, electrophysiology is significantly outside of our expertise but we invite the reviewer to consult the literature regarding this differentiation, including Thoma et al 2012 and more recently Vainorius et al 2023. We aim to demonstrate here that Ngn2 can be driven downstream of our inducible systems to induce differentiation and make no claims regarding the physiology of the differentiated cells.

35. Reviewer 3 asked for the off-rate of our systems which we have now tested and added to Supplemental Figure 2.

36. As requested by Reviewer 3, we have compared our system to the classic TET-On system in Supplemental Figure 10.

37. Reviewer 3 says "[t]he authors claim is to develop 3 different orthogonal systems for controlling gene activity in stem cells. However, 2 out of 3 approaches performed very poorly" and asks how these tools can be useful in developmental biology. By our count, we demonstrate 5 different systems (4OHT, ABA, GZV, synNotch, and SNIPR). We agree with the reviewer that at least one of the systems, synNotch, performs poorly in our implementation (although not in all implementations, cf SynPL). We also agree with the reviewer that at least the GZV system performs poorly in neuronal differentiation because it cannot produce sufficient Ngn2 to induce differentiation. However, as all systems perform well in TagBFP production, we think their relative usefulness is dependent on the user's intent. For example, a neighboring lab adopted our GZV system in order to produce Wnt and DKK in a synthetic developmental study that is currently in press. In cases where signals will be amplified, or smaller proteins will be produced, this system that failed to work in differentiation still proved very useful. To address the reviewer's concern, we have added a paragraph to the Discussion comparing our system to existing systems, and we have attempted to highlight throughout how each systems' strengths and weaknesses position it for use in particular settings.

38. Reviewer 3 noted that WT mESCs were not the correct controls in (now) Figure 5 and 6. We agree, and in fact used cells as described with the output circuit layer, but our terminology failed to demonstrate this. We have updated our terminology throughout.

© 2025. Published by The Company of Biologists under the terms of the Creative Commons Attribution License (https://creativecommons.org/licenses/by/4.0/).

Resubmission

First decision letter

MS ID#: dev.204505

MS TITLE: Small molecule and cell contact-inducible systems for controlling expression and differentiation in mouse embryonic stem cells

AUTHORS: Sarah S. Soliman; Devan H. Shah; Hana El-Samad; Zara Y. Weinberg

I am happy to tell you that your manuscript has been accepted for publication in Development, pending minor modifications based on feedback from reviewer 2 and our standard publication integrity checks. It was accepted on 31 Mar 2025. Where referee reports on this version are available, they are appended below.

Reviewer 1

This is a re-review. The authors have responded appropriately to my comments.

The way the authors structured their responses to reviewers made it very difficult for reviewers to assess what had been done. The normal thing to do is to respond to each reviewer's point in turn, so that a reviewer can look at what they said and assess what has been done about each point. Please do this in future; reviewers are giving up their time free to help you and the journal, and making it more difficult for them to align their original comments with your responses increases their workload unnecessarily. Thanks.

Reviewer 2

Advance summary and potential significance to field

In this manuscript, Soliman et al. implement several synthetic biology tools in mouse embryonic stem cells (mESCs). Some had previously been shown to be functional in mESCs (ERT2/4-OHT, ABI/PYL/abscisic acid, and synNotch), whereas others are characterized in this cell type for the first time (NS3/grazoprevir, SNIPRs). All had been previously developed and characterized in other cell types.

The authors demonstrate how these tools can be used not only to overexpress fluorescent reporters, but also to drive neuronal differentiation of pluripotent cells in non-permissive conditions by selectively inducing the pro-neural transcription factor Neurogenin2 (the neurogenic potential of which in pluripotent cultures was previously reported).

This article represents an important stepping stone in the expanding field of synthetic developmental biology, demonstrating functionality of several synthetic biology technologies in mESCs for the first time, and comparing different tools, thus encouraging researchers who make use of these cell lines to incorporate these tools into their research.

Comments for the author

The authors did a commendable job of improving the manuscript since their original submission, both in terms of scope and of clarity. The new Figure 2 in particular is a very valuable addition.

Most of the points I raised have been addressed.
There are three main points I feel still need addressing.

© 2025. Published by The Company of Biologists under the terms of the Creative Commons Attribution License (https://creativecommons.org/licenses/by/4.0/).

1- What are BFP levels in wild-type cells vs UAS-BFP cells? This has important implications for users wishing to induce a functional transgene.
2- I have a problem with the scoring of "neurons" in Figure 5 based on "above-baseline Tubb3 expression". The levels of Tubb3 in neurons, as shown in Figure 5B, are well above baseline, and there is a clear discrepancy between IF and quantitative data because of this.
3- In their response to reviewers, the authors explain that the main benefit of this work to the Developmental Biology community will consist of the DNA constructs which have been generated. Rather than the following sentence: "All plasmids will be available on Addgene.", I would request that authors deposit the plasmids with Addgene, and share a supplemental table with plasmid information and Addgene codes, to streamline resource sharing following publication. Addgene allows for plasmid distribution to be embargoed until publication.

Specific comments:

Lines 131-133 and Figure S2A-C
Please also include tagBFP and mCherry levels in untransduced wild-type cells in all three panels:
"In the absence of inducer, UAS-BFP cells and each transcription factor-expressing subline showed minimal BFP expression or leakage at baseline (Figure S2C)."
The panel shows the three systems have somewhat comparable baseline tagBFP levels to the UAS-BFP controls. It does not however show how leaky all these cell lines are compared to wild-type, which is very important for anyone wishing to induce a functional transgene in place of BFP.

Figure S2E
It would be great to include the "no drug" data in these panels if it is already available.

Figure S3 legend
"Each graph displays the absolute stainingâ€¦"
Please clarify what this means. Median level of fluorescent signal across 3 biological replicates? Something else?

Figure S4 B,C
It is unclear to me what the graph is displaying. Based on the main text and the legend, I was expecting a single set of bars, indicating what proportion of ESCs are capable of differentiating into EpiLCs in each respective condition. What is the second set of bars representing?
It would be helpful (to me at least!) to make this clearer.

Lines 183-190
Mention the possibility that the effect may not be on differentiation potential, but on differentiation speed.

Lines 194-195
Please include appropriate references for the markers used.

Figure S4E
As above, I'm not sure what these graphs are displaying. I was expecting two sets of bars, not three, based on the main text and legend (efficiency of differentiation to ectoderm, efficiency of differentiation to XEN).

Figure 3 and lines 269-270
I find the term "No Notch" confusing. I initially thought these were knockouts for one or more of the Notch genes. "No receptor" would be easier for me to understand.

Figure 3 and S5C
Could it be made explicit in the text that the levels of mCherry are very different between senders and receivers?
The receivers look "negative" compared to the senders.
It would be useful to discuss whether this may be due to poor tolerance of cells for these receptors or whether there may be issues with transgene delivery.
For panel 3C, could the authors also include levels of BFP in wild-type cells? Understanding the baseline leakiness of the system is important for users who wish to induce a functional transgene.

© 2025. Published by The Company of Biologists under the terms of the Creative Commons Attribution License (https://creativecommons.org/licenses/by/4.0/).

Figure S5C
Would it be possible to make it clear that the "mESC senders" are in fact wild-type, and not sending any signal (I find the term senders confusing)? E.g. by calling them "No ligand control mESCs" or something similar. (I was expecting to see BFP signal in the NbALFA-SNIPR Receivers until I realised these were wild-type mESCs.)

Line 323
Comment as to why mock-transfected cells in SFES do not form neurons.
I appreciate a theoretical answer to this question has been given in the response to reviewers, but it should also be addressed in the main text, as most readers will be puzzled by this result, and would expect an explanation.

Line 330 and 338
Please clarify that these cells were cultured in 2i in main text.

Figure 5 and related main text (lines 325-361)
I disagree with the interpretation of "Tubb3-positive = neuron".
Thanks to the authors' new Figure S6A, it is clear there is a continuum of Tubb3 expression, and that the author's inducible systems increase levels of Tubb3.
But this does not mean, for example, that they are generating >70% neurons in ABA (Fig.5C): this is made clear by the IF for ABA in Figure 5B, where only two neurons are observed in a field of 50-odd cells.
The quantification shown in Figure 5C would be more accurate (and useful), if the authors gated for positive cells based on levels seen in actual neurons, rather than gating for anything above baseline, which might be a cell that is starting to differentiate rather than a neuron.

Reviewer 3: no comments. Concerns are addressed.

© 2025. Published by The Company of Biologists under the terms of the Creative Commons Attribution License (https://creativecommons.org/licenses/by/4.0/).
